# Influence of 16S rRNA target region on the outcome of microbiome studies in soil and saliva samples

**DOI:** 10.1038/s41598-020-70141-8

**Published:** 2020-08-12

**Authors:** Ana Soriano-Lerma, Virginia Pérez-Carrasco, Manuel Sánchez-Marañón, Matilde Ortiz-González, Victoria Sánchez-Martín, Juan Gijón, José María Navarro-Mari, José Antonio García-Salcedo, Miguel Soriano

**Affiliations:** 1 Department of Physiology (Faculty of Pharmacy, Campus Universitario de Cartuja), Institute of Nutrition and Food Technology “José Mataix”, University of Granada, 18071 Granada, Spain; 2 GENYO. Centre for Genomics and Oncological Research: Pfizer/University of Granada/Andalusian Regional Government, PTS Granada, 18016 Granada, Spain; 3 Microbiology Unit, Biosanitary Research Institute IBS.Granada, University Hospital Virgen de las Nieves, 18014 Granada, Spain; 4 Department of Soil Science and Agricultural Chemistry, University of Granada, 18071 Granada, Spain; 5 Center for Intensive Mediterranean Agrosystems and Agri-Food Biotechnology (CIAIMBITAL), University of Almeria, 04001 Almería, Spain; 6 Department of Periodontics, School of Dentistry, University of Granada, Granada, Spain

**Keywords:** Microbiology, Microbial communities, Metagenomics, Microbiome

## Abstract

Next generation sequencing methods are widely used in evaluating the structure and functioning of microbial communities, especially those centered on 16S rRNA subunit. Since Illumina Miseq, the most used sequencing platform, does not allow the full sequencing of 16S rRNA gene, this study aims to evaluate whether the choice of different target regions might affect the outcome of microbiome studies regarding soil and saliva samples. V1V3, V3V4, V4V5 and V6V8 domains were studied, finding that while some regions showed differences in the detection of certain bacterial taxa and in the calculation of alpha diversity, especially in soil samples, the overall effect did not compromise the differentiation of any sample type in terms of taxonomic analysis at the genus level. 16S rRNA target regions did affect the detection of specific bacteria related to soil quality and development, and microbial genera used as health biomarkers in saliva. V1V3 region showed the closest similarity to internal sequencing control mock community B, suggesting it might be the most preferable choice regarding data reliability.

## Introduction

Over the last decade, there has been a rapid rise in the development of microbiome studies because of the understanding of microbial communities as one of the main factors involved in environmental processes and in human health and disease. The affordability, reliability and high-throughput yield of data provided by next-generation sequencing (NGS) technologies has led to the substitution of classical approaches, such as culture-based methods, in the characterization of microorganisms^1^.

Depending on the objective of the research, amplicon sequencing or whole metagenome sequencing can be used to gain insight into microbial structural and functional patterns. Sequencing analysis of 16S rRNA subunit is the most widely used and cost effective strategy to discern the structure of the microbiome in all sorts of environments^2,3^. 16S rRNA is a highly conserved and universal gene, whose sequence is about 1,500 bp long and consists of nine hypervariable regions (V1–V9) separated by conserved segments, working the latter as target sequences for primer design^4^. 16S rRNA sequence has been fully characterized in a great number of bacterial species and strains, and a wide variety of primers have been designed to offer a broad applicability in the analysis of microbiome structure^2,5^, especially through the use of NGS platforms such as Illumina Miseq^3^.

Since Illumina Miseq does not allow the full sequencing of 16S rRNA (up to 600 bp), its hypervariable regions can be used individually or can be combined to assess the structure of bacterial communities^6^. It has been shown that both the use of different primers and regions affects the outcome of microbiome studies^5,7–11^, and thus, such factors should be taken into consideration when designing a sequencing analysis. There are a considerable number of studies analyzing the effect of primers choice in the profiling of microbiome communities. It is known that the analysis of the human microbiome and microbial alpha diversity in stool samples differs if V3V4 or V4V5 region is targeted^7^, while other studies show only slight differences when selecting either V1V3, V3V4 or V4 regions in the same type of samples^3^. Comparable studies have been developed in environmental (water) samples, showing that V4 region is more suitable to achieve accurate sequence assignment in the Bacteria domain, along with increased coverage^10^. Albertsen et al.^9^ found differences in bacterial taxa distribution between V1V3, V3V4 and V4 regions, but similar alpha diversity. However, there is still a gap in our knowledge in this area, especially as far as highly diverse samples are concerned. Studies addressing the detailed impact of 16S rRNA regions on the profiling of bacterial communities in both environmental and biological samples have not been published to date, and nor have comprehensive analysis including the use of combined regions such as V1V3, V3V4, V4V5 and V6V8, the most commonly used to increase taxonomic accuracy in amplicon sequencing. Additionally, the existing studies lack analysis of the influence of 16S rRNA regions on the determination of statistical differences between samples, being specifically focused on the assessment of general microbial patterns. In this study, soils were selected as representatives for highly diverse environmental samples^12^ and so were saliva samples as its biological counterparts^13^, since no scientific articles analyzing the effect of 16S rRNA target regions have been published regarding those samples types.

Soils are complex ecosystems with a huge variability in environmental characteristics and microbial community structure, both at the global and local scale^14^. A plethora of research has documented the relationships between microbial traits and soil properties, being most of them focused on single parameters such as pH, temperature, vegetation, texture, soil water content, nitrogen, organic matter or pollutants^15–25^. Nevertheless, given the complex interrelationships between soil microbes and site conditions, it is unlikely that their variation will stem from a single influence or a small set of factors^14^. Few studies consider the soil as a whole from a pedological point of view, which may be useful in the search of unifying principles in soil microbial ecology^14,22^. In a previous paper^26^ we established that soil genesis or development (pedogenesis) may be considered as an integrating concept summarizing the joint influence of environmental factors (parent material, vegetation, topography, and climate) and historical contingences. Eight soil typologies with different development and management were selected as representative for Mediterranean calcimorphic environments, widely extended in Mediterranean climate-type areas and, in general, in semiarid regions around the world. These soils represented a pedogenic gradient from the less-developed soils (Leptosols) to more developed soils (Luvisols). Our results showed that the relative abundance of *Acidobacteria*, *Canditate division WPS-1*, and *Armatimonadetes* decreased whereas that of *Actinobacteria*, *Bacteroidetes*, and *Proteobacteria* increased from low developed to highly developed soils. This pattern in bacterial distribution was also correlated with soil quality indicators such as organic C, water-stable aggregates, porosity, moisture, and acidity. Other works also found similar correlations patterns with soil quality indicators; namely, *Acidobacteria* correlated to organic carbon and pH^20^, with some subgroups showing a positive correlation, such as *GP4* and *GP6*, and others being negatively correlated, such as *GP1*, *GP7*, or *Actinobacteria* phylum^20,25^*. Acidobacteria*, *Gemmatimonadetes* and *Verrucomicrobia* also showed negative correlations to nitrogen^23^.

On the other hand, salivary microbiome research is becoming increasingly relevant since it allows a non-invasive assessment of disease status, progression and therapeutic efficacy through microbial biomarker discovery^27^. It is widely known that saliva is among the human biological niches with highest alpha diversity and lowest beta diversity^13,28,29^, even between different ethnicities^29^, four healthy oral types, and its association with lifestyle factors, have been described to date^30^. It has been shown that a decreased alpha diversity, as well as alterations in certain taxa such as opportunistic Gammaproteobacteria or pathogenic families *Enterobacteriaceae* and *Enterococcaceae*, among others, are associated with autoimmune and metabolic disorders, cancer and immunodeficiency states^30^. Microbial indicators have also been described in relation to treatment response in the case of aggressive periodontitis^31^, emphasizing its importance to predict therapeutic outcome, as well as to design preventive, diagnostic and prognostic tools. Therefore, the objective of this study is to evaluate the impact of the selected hypervariable regions on the statistical differentiation of soil samples collected along a pedogenic gradient^26^ and saliva samples from healthy individuals with different oral types^28,32,33^. It will also aim to discern which of the selected target regions yields the most reliable data.

## Results

### Structure of microbial communities at the phylum and genus level considering each region in soil and saliva samples

Sequencing of 16S rRNA gene amplicons with Illumina MiSeq resulted in a total of 5308825 sequences after bioinformatic processing (1208831 reads for V1V3, 1250262 for V3V4, 1497786 for V4V5 and 1351946 for V6–V8), with an average length of 492 bp for V1V3, 457 bp for V3V4, 412 bp for V4V5 and 438 bp for V6V8 (447 bp overall mean length). The first quality-filtering (see “Methods”) had the greatest impact on V1V3 region, which showed the highest percentage of removed sequences in soil and saliva samples; at this point, soil sequences were reduced by an additional 10% when compared to saliva samples (Table 1). The second quality-filtering showed a similar influence on all domains and both sample types (sequences were reduced by approximately 1%). Lastly, removal of chimeric reads resulted in a reduction of 10–30% in the number of sequences in soil and saliva samples, with the highest impact on V3V4 domain, whose sequences were reduced by 30% in soil samples. The smallest overall percentage of removed sequences in soil samples was found in V4V5 region, while in saliva samples was noticed in V3V4 region; V1V3 region showed the greatest removal of sequences in both sample types. Retained sequences increased progressively about 5–6% between 16S rRNA regions, although in different order in both soil and saliva samples, with a maximum difference of 12% and 16% respectively (Table 1). A detailed analysis of sequences along the bioinformatic procedure is provided in Supplementary File 1 for each sample.

**Table 1:** Analysis of sequences along the bioinformatic procedure in all 16S rRNA regions and each sample type (soil, saliva).

Soil					% of retained sequences				
Sample	Raw sequences	After 1st step	After 2nd step	After 3rd step	Sample	Raw sequences	After 1st step	After 2nd step	After 3rd step
V1V3 (n = 32)	1922458	1311295	1293625	1013137	V1V3	1922458	68.21	67.29	52.70
V3V4 (n = 32)	1491453	1323478	1310614	876815	V3V4	1491453	88.74	87.87	58.79
V4V5 (n = 32)	1696973	1370970	1365861	1093159	V4V5	1696973	80.79	80.49	64.42
V6V8 (n = 32)	1480504	1206998	1197464	858655	V6V8	1480504	81.53	80.88	58.00
					% of removed sequences				
					V1V3	100	31.79	0.92	14.59
					V3V4	100	11.26	0.86	29.09
					V4V5	100	19.21	0.30	16.07
					V6V8	100	18.47	0.64	22.88

Saliva					% of retained sequences				
Sample	Raw sequences	After 1st step	After 2nd step	After 3rd step	Sample	Raw sequences	After 1st step	After 2nd step	After 3rd step
V1V3 (n = 11)	368192	285585	276884	195694	V1V3	368192	77.56	75.20	53.15
V3V4 (n = 11)	534680	484986	481163	373447	V3V4	534680	90.71	89.99	69.84
V4V5 (n = 11)	626050	492403	477859	404627	V4V5	626050	78.65	76.33	64.63
V6V8 (n = 11)	796496	674591	667415	462041	V6V8	796496	84.69	83.79	58.01
					% of removed sequences				
					V1V3	100	22.44	2.36	22.05
					V3V4	100	9.29	0.72	20.15
					V4V5	100	21.35	2.32	11.70
					V6V8	100	15.31	0.90	25.78

The reduction in the number of sequences and the percentage of retained and removed sequences after three bioinformatic steps is detailed. The first step includes a quality-filtering removing sequences according to criteria regarding ambiguous bases, homopolymers and length; the second step involves the removal of poorly aligned sequences against the reference database; lastly, the third step includes the removal of chimeric reads.

**Figure 1:**
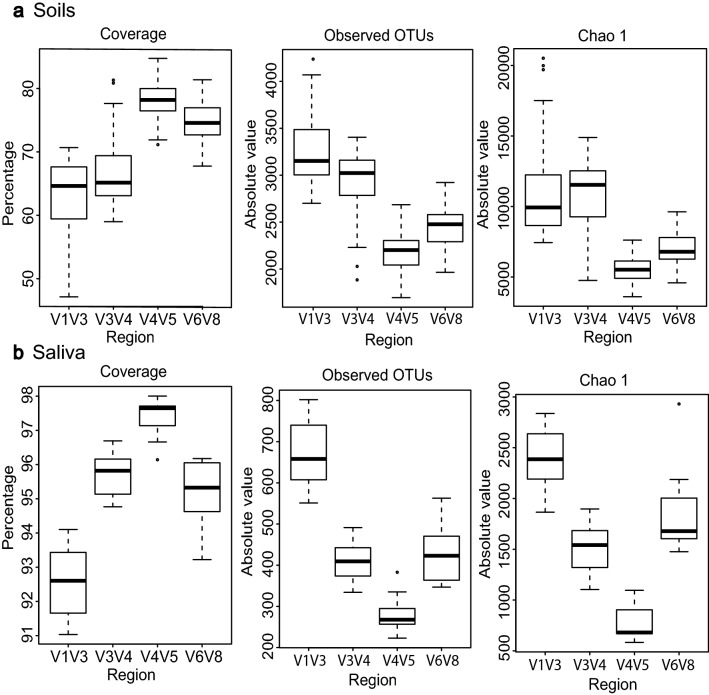
Alpha diversity variables in each 16S rRNA region considering soil (**a**) and saliva samples (**b**). Coverage, number of observed OTUs and richness index Chao1 were calculated defining OTU at 3% of dissimilarity by opticlust method (Mothur v1.43.0 software, University of Michigan Medical School, Ann Arbor, MI, USA). (**a**) Coverage, number of observed OTUs and richness index Chao1 in each 16S rRNA region considering all soil samples (n = 32). (**b**) Coverage, number of observed OTUs and richness index Chao1 in each 16S rRNA region considering all saliva samples (n = 11).

Technical validation was next implemented to assess reproducibility (Supplementary Fig. S1). Permutational multivariant analysis of variance (PERMANOVA) was used to determine statistical differences between technical replicates, while principal coordinate analysis (PCoA) was performed to assess beta diversity. No statistical differences were found in multivariant PERMANOVA test (Pseudo-F = 0.75439, p = 0.5155), and accordingly, PCoA also showed low beta diversity between technical replicates (Supplementary Fig. S1). Both extraction and PCR negative controls showed no significant amplification in agarose gels, yielding a small number of sequences. No common OTUs with considerable abundances in negative controls and samples were found. Within community alpha diversity was assessed in both sample types and each 16S rRNA region. To minimize sample size-induced bias between the datasets, we rarefied all samples by sub-sampling at the lowest number of obtained sequences (6589 sequences). The number of observed OTUs and richness index Chao1 were higher for V1V3 and V3V4 in soil samples, along with its consequent decrease in coverage; however, in saliva samples, V3V4 region showed lower values compared to soils, even below those of V6V8 region (Fig. 1). As for the rest of alpha diversity parameters, V1V3 showed the highest values in soil and saliva samples (Supplementary Fig. S2); V4V5 region showed the lowest values in soil samples, while in saliva, InvSimpson and Pielou indexes were lower for V6V8 region compared to V4V5 (Supplementary Fig. S2).

**Figure 2:**
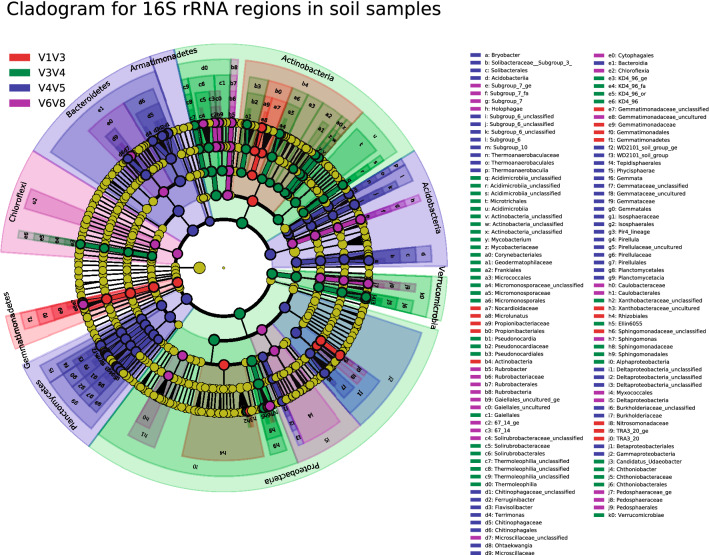
Linear discriminant analysis Effect size (LEfSe): cladogram for differentially distributed taxa (p < 0.01, LDA > 3.5) between 16S rRNA regions in soil samples (n = 32). Taxonomic features are represented in a hierarchical structure, with higher phylotypes oriented towards the inner part of the plot. Taxa showing significant differences between 16S rRNA domains are coloured according to the most abundant class (red for VIV3, green for V3V4, blue for V4V5, purple for V6V8, yellow for non-significant). Statistical analysis and representation was carried out using Python 3.7.6. For further information on taxa showing statistical differences between 16S rRNA regions in soil samples (p < 0.05, LDA > 2), see Supplementary file 2.

**Figure 3:**
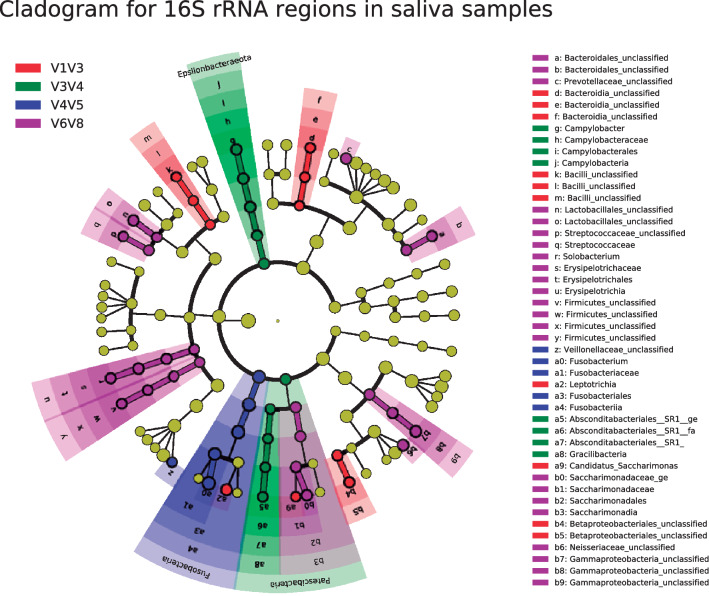
Linear discriminant analysis Effect size (LEfSe): cladogram for differentially distributed taxa (p < 0.05, LDA > 2) between 16S rRNA regions in salival samples (n = 11). Taxonomic features are represented as in Fig. 2.

Taxonomic analysis at the phylum level detected differences in relative abundance among all four 16S rRNA regions. In soil samples, the taxa detected by V1V3 and V3V4 regions were similar, in order of abundance: *Proteobacteria, Actinobacteria, Acidobacteria, Bacteroidetes, Planctomycetes, Gemmatimonadetes*, *Chloroflexi* and *Verrucomicrobia*. V6V8 region yielded a slightly different pattern when compared to V1V3 and V3V4 regions, with *Proteobacteria, Actinobacteria, Acidobacteria, Bacteroidetes* being the most abundant taxa, followed by *Chloroflexi, Verrucomicrobia, Gemmatimonadetes* and *Planctomycetes*. Lastly, the V4V5 region showed the most dissimilar pattern, being *Acidobacteria, Proteobacteria, Bacteroidetes, Planctomycetes, Actinobacteria, Gemmatominadetes, Verrucomicrobia* and *Chloroflexi* the most abundant taxa (Supplementary Fig. S3). Phyla showing statistical differences between 16S rRNA regions in soil samples were assessed through LEfSe analysis, confirming an increased abundance of *Armatimonadetes*, *Acidobacteria, Planctomycetes* and *Bacteroidetes* for V4V5 region (Fig. 2, Supplementary File 2). In the case of saliva samples, all domains showed similar microbial composition for the most abundant phyla (*Firmicutes, Proteobacteria, Bacteroidetes, Fusobacteria* and *Actinobacteria*), except for *Fusobacteria*, whose abundance was increased in V4V5 region (Supplementary Fig. S4). LEfSe analysis confirmed differences in *Fusobacteria* phylum for V4V5 region (Fig. 3, Supplementary File 3).

A principal component analysis (PCA) was performed on soil and saliva samples considering each 16S rRNA region and including the most abundant and differently distributed phyla between samples. In the case of soil samples (Supplementary Fig. S5), PCA accounted for around 60% of the bacterial variation in all 16S rRNA regions considering the first two principal components, while 80% was reached in saliva samples (Supplementary Fig. S6). A similar distribution pattern could be observed in all 16S rRNA regions for soil and saliva samples. Considering soil samples, *Proteobacteria* and *Bacteroidetes* were associated with soils S3, S5, S6 and S7, and inversely correlated to *Actinobacteria, Firmicutes, Chloroflexi, Nitrospirae, Armatimonadetes, Gemmatimonadetes, Cyanobacteria, Acidobacteria, Planctomycetes* and *Verrucomicrobia* which in turn were associated with soils S1, S2 and S4. S8 showed an intermediate microbial composition, being therefore situated in the middle of the plot (Supplementary Fig. S5). On the other hand, saliva samples were separated in two groups or oral types along the X axis, associated either with *Fusobacteria*, *Bacteroidetes* and *Actinobacteria* or *Proteobacteria* (Supplementary Fig. S6).

**Figure 4:**
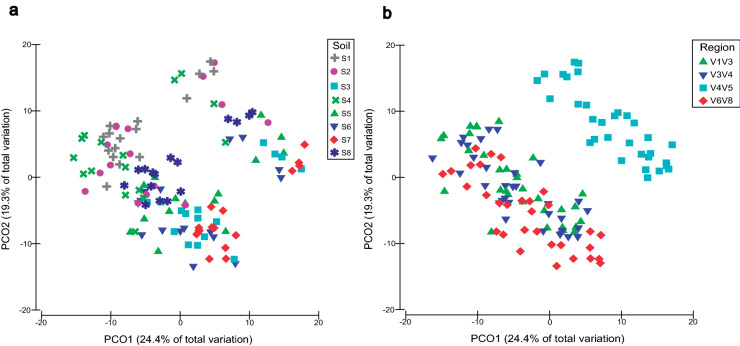
Principal coordinate analysis (PCoA) based on Bray–Curtis distances. Plots for the bacterial genera with a relative abundance higher than 0.05% in soil samples. Samples are represented by coloured symbols according to the legend. (**a**) Different types of soil with their respective replicates are considered regardless of 16S region (n = 128). (**b**) Different 16S regions are considered regardless of the type of soil (n = 128). PRIMER e Permanova + (PRIMER-E Ltd, Plymouth, UK) was used in the implementation of the statistical analysis.

**Figure 5:**
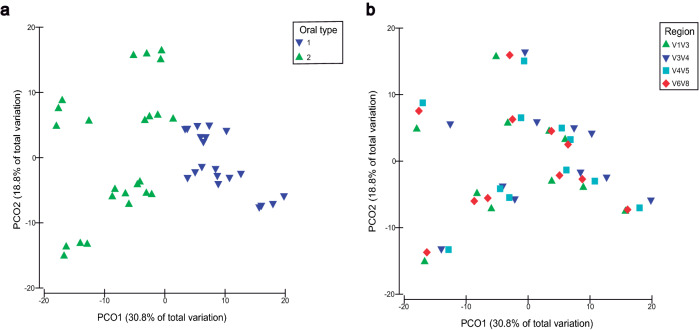
Principal coordinate analysis (PCoA) based on Bray–Curtis distances. Plots for the bacterial genera with a relative abundance higher than 0.05% in saliva samples. Samples are represented by coloured symbols according to the legend. (**a**) Different oral types are considered regardless of 16S region (n = 44). (**b**) Different 16S regions are considered regardless of the respective saliva sample (n = 44). PRIMER e Permanova + (PRIMER-E Ltd, Plymouth, UK) was used in the implementation of the statistical analysis.

At the genus level, the maximum number of detected genera which showed a relative abundance higher than 0.05% in soil samples was 257 (V1V3 region), while in saliva 77 were detected (V1V3 region). Particularly, in soil samples the V3V4, V4V5 and V6V8 regions detected 237, 249 and 238 genera respectively, being in saliva 51, 59 and 59 detected by the same regions. Principal coordinate analysis (PcoA) was performed on soil and saliva samples considering all genera showing a relative abundance higher than 0.05%. In soil samples (Fig. 4), PcoA accounted for 43.7% of bacterial variation considering the first two principal coordinates. Soils S1–S8, with its respective replicates, clustered differentially along the X axis, regardless of the sequenced region. Soils S1, S2 and S4 were located on the top left part of the plot, and the same occurred to S3, S5, S6 and S7 on the bottom right part of the graphic, with S8 situated in the middle (Fig. 4a). When analyzing the sequenced region, V1V3, V3V4 and V6V8 domains clustered together, while V4V5 did it separately (Fig. 4b). In the case of saliva samples (Fig. 5), PCoA represented 49.6% of bacterial variation considering the first two principal coordinates. Saliva samples clustered in two different groups or oral types, irrespective of the sequenced region (Fig. 5a). Unlike soil samples, when analyzing the target regions, all of them clustered together (Fig. 5b).

Principal component analysis (PCA) at the genus level was also carried out in both sample types to corroborate results obtained by principal coordinate analysis (PCoA). A differentiation of soils S1–S8 was also observed along the X axis regardless of the target region, with soils S1, S2 and S4 situated in the bottom right corner of the plot, soils S3, S5, S6 and S7 in the top left part of the graphic, and S8 in the middle (Supplementary Fig. S7a). Similarly, in soil samples V4V5 region clustered separately compared to V1V3, V3V4 and V6V8 domains (Supplementary Fig. S7b). Considering saliva samples, two oral types were also observed irrespective of the target region (Supplementary Fig. S8a), with all domains clustering together (Supplementary Fig. S8b). The identification of the 20 main variables contributing to the first two principal components in the PCA was next performed on both sample types to identify bacterial genera related to soil development and oral types (Supplementary Table S1). In the case of soil samples (Supplementary Fig. S7a), variables associated with highly developed soils (upper left corner of the plot) and low-developed soils (bottom right corner of the plot) were identified, as well as those associated with oral type 1 (left side of the plot) and 2 (bottom right corner of the plot) in saliva samples (Supplementary Fig. S8a).

### Influence of 16S rRNA region on the outcome of microbiome studies involving soil and saliva samples

To determine the effect of the target region on the detection of statistical differences between types of soil and saliva oral types, permutational multivariant analysis of variance (PERMANOVA) was performed considering alpha diversity indexes and bacterial genera as variables. As far as alpha diversity indexes are concerned, multivariant PERMANOVA test revealed differences between soils and oral types, as well as differences between target regions. Regarding the taxonomic analysis, statistical differences were found between soils and oral types, as well as between target regions (Table 2). In order to determine the factor levels for which statistical differences in taxonomic variables were detected, multivariant PERMANOVA pairwise test was then carried out for each factor in both sample types. With regard to soil samples, all types of soils were statistically different from each other, as well as all target regions. As for saliva samples, statistical differences were detected between the two oral types, while only V4V5 region was statistically different from V1V3 and V6V8 domains (Supplementary Table S2). A summary of means and standard deviations of diversity and taxonomic variables included in the analysis in both samples types is available in Supplementary File 4 (soils) and Supplementary File 5 (saliva).

**Table 2:** Multivariant PERMANOVA test considering alpha diversity indexes and bacterial genera as variables in soil (n = 128) and saliva samples (n = 44).

Soil Samples	Pseudo-F	p (perm)	Sig	Saliva Samples	Pseudo-F	p (perm)	Sig
**Statistical analysis at the genus level**							
Multivariant PERMANOVA test							
Region	43.943	0.0001	*	Region	1.7444	0.0384	*
Soil	15.331	0.0001	*	Oral type	10.783	0.0001	*
Region × soil	0.22478	1	n.s	Region × oral type		1	n.s
**Statistical analysis of microbial diversity**							
Multivariant PERMANOVA test							
Region	4.7444	0.0002	*	Region	7	0.0019	*
Soil	74.445	0.0001	*	Oral type	3	0.0001	*
Region × soil	0.8089	0.7239	n.s	Region × oral type	21	0.3914	n.s

PRIMER e Permanova + (PRIMER-E Ltd, Plymouth, UK) was used in the implementation of the statistical analysis.

Interestingly, when analyzing the differences between target regions within each type of soil, V1V3 and V3V4 domains did not show statistical differences in any soil except for S7 and S8; the other regions differed in soils S1–S8 (Supplementary Table S3). The differences detected between types of soil within each target region also varied; V4V5 and V6V8 domains yielded a similar pattern and detected statistical differences between S3–S7 and S6–S7, while V1V3 and V3V4 regions did not. V4V5 detected differences between S1–S2, while the other regions failed in their detection (Supplementary Table S4). Conversely, in saliva samples, the differences between regions were not affected by oral types and neither were differences detected between oral types affected by the different target regions (Supplementary Table S5).

In order to individually evaluate statistical differences, univariant PERMANOVA test was performed on soil and saliva samples considering alpha diversity indexes and bacterial genera as variables (Supplementary File 6 for soil samples and Supplementary File 7 for saliva samples). In this regard, LEfSe analysis was also carried out to expand statistical assessment up to the phylum level, and to identify the specific 16S rRNA regions for which statistical differences were detected in each microbial taxa (Supplemenary file 2 for soil samples and Supplementary File 3 for saliva samples). All diversity variables varied between soils S1–S8 and between 16S rRNA regions except for Chao1, which did not differ between any type of soil (Supplementary file 6). At the genus level, LEfSe analysis detected statistical differences between 16S rRNA regions in a wide variety of microbial taxa, such as *Terrimonas*, *Gemmataceae_uncultured*, *Armatimonadetes_uncultured_ge* or *Solirubrobacter* (Fig. 2, Supplementary file 2).

In relation to saliva samples, all diversity variables differed between 16S rRNA regions while any yielded statistical differences between oral types, except for InvSimpson (Supplementary File 7). LEfSe analysis showed fewer statistical differences in bacterial genera between 16S rRNA regions compared to soils (Fig. 3, Supplementary File 3). Namely, *Campylobacter, Solobacterium, Erysipelotrichia, Fusobacterium* and *Leptotrichia* were differently detected by 16S rRNA target regions in saliva samples (Fig. 3).

The majority of contributing bacteria in the PCA (Supplementary Table S1) related to soil development showed statistical differences between target regions in soil samples (Supplementary File 2). Some illustrative examples were plotted in Supplementary Fig. S9: *Terrimonas*, *Nocardioides*, *Gemmataceae_uncultured* and *Armatimonadetes_uncultured_ge*. As far as saliva samples are concerned, slighter differences in PCA-contributing bacteria between all target regions were found (Supplementary File 3). By way of illustration, the genera which might represent both oral types (*Family_XIII_ge* and *Catonella* representing oral type 1 and *Neisseria* representing oral type 2) were plotted, showing that all domains similarly assessed changes between oral types (Supplementary Fig. S10).

### Mock community B as an internal control for 16S rRNA sequencing

Resemblance matrixes performed on mock community B sequenced with the different 16S rRNA regions revealed the closest similarity for V1V3 and V3V4 domains and mock community theoretical composition at the genus level, while V4V5 and V6V8 regions yielded the lowest percentage of similarity (Table 3) (Supplementary Fig. S11).

**Table 3:** Resemblance matrix showing the percentage of similarity between mock community B sequenced with V1V3, V3V4, V4V5 and V6V8 target regions and its theoretical composition.

	Theoretical composition	Mock_V1V3	Mock_V3V4	Mock_V4V5	Mock_V6V8
**Theoretical composition**					
Mock_V1V3	72.085				
Mock_V3V4	71.488	92.596			
Mock_V4V5	69.942	90.18	90.065		
Mock_V6V8	68.151	92.045	91.238	93.795	

The analysis was implemented in PRIMER e Permanova + (PRIMER-E Ltd, Plymouth, UK), considering Bray–Curtis distances.

## Discussion

In the present study, differences in bacterial community structure were assessed in soil and saliva samples according to the use of different 16S rRNA regions, considering V1V3, V3V4, V4V5 and V6V8 hypervariable domains, as well as the influence of sample type (soils and saliva) on domain-dependent effects. Analysis of resemblance between 16S rRNA regions and mock community sequencing control was also carried out in order to determine which primer set yielded the most reliable data. This study therefore aims to provide some guidance as to which 16S rRNA region should be chosen depending on the objective of the research and the sample type.

16S rRNA regions affected differently to soil and saliva samples, being the impact much more evident in soils. Firstly, the capability to detect certain phyla differed between target regions. In soil samples, V1V3, V3V4 and V6V8 regions similarly detected the most abundant phyla, while V4V5 differed. Particularly, V4V5 domain was biased towards an increased abundance of *Armatimonadetes*, *Acidobacteria, Planctomycetes* and *Bacteroidetes* (Fig. 2). To date, few scientific articles have been published in relation to the influence of 16S rRNA target regions on environmental samples, and none was conducted on soils, the most diverse ecosystem^12^. Wetland sediments and sludge communities have been mainly used instead, demonstrating a strong influence of the 16S rRNA domain on the assessment of microbiome structure and the detection of specific taxa^9,11^. However, the existing studies are focused on analyzing the first (V1V3, V3V4, V4) or last (V4, V6V8, V7V8) 16S rRNA regions and do not consider the combined domain V4V5, which provides higher taxonomic accuracy compared to V4 single domain^34^. As already discussed, decreases in the abundance of *Armatimonadetes* and *Acidobacteria*, along with increases in the abundance of *Bacteroidetes,* are associated with soil development^26^. Similarly, our study showed that V4V5 region detected an increased abundance of *Fusobacteria* in saliva samples (Fig. 3). Negative correlations have been described for *Fusobacteria* phylum in the salivary microbiome in the case of pancreatic cancer risk^35^, as well as a decreased relative abundance in HIV seropositive compared to seronegative individuals^36^.

Despite the fact that V4V5 domain was skewed regarding the detection of certain phyla, its capacity for evaluating the structure of microbial communities in soils and saliva samples was not affected at the phylum and genus level. A similar distribution of both sample types could be observed in principal component analysis (PCA) in all 16S regions at the phylum level (Supplementary Figs. S5 and S6). Specifically, soils were separated along a pedogenic gradient in the X axis, with highly developed soils S3, S5, S6 and S7 being microbially different from low-developed soils S1, S2 and S4 (Supplementary Fig. S5)^26^. Soil development increases from S1 to S8 but external factors such as soil management may also affect microbial community structure^26^. Thus, although being classified as the most developed soil, S8 showed an intermediate microbial composition between developed and undeveloped soils^26^. Similarly, two oral types could be differentiated in all 16S rRNA regions in saliva samples at the phylum level (Supplementary Fig. S6). Comparable results were obtained when performing principal coordinate analysis (PCoA) at the genus level in both sample types. A pedogenic gradient could also be observed in soil samples, regardless of the target region, with soil development increasing along the X axis (Fig. 4a). Samples sequenced under V1V3, V3V4 and V6V8 primer conditions were grouped together, while those belonging to V4V5 domain clustered separately (Fig. 4b). These results confirm that, while correctly evaluating changes in microbial composition between samples, the V4V5 domain presented a different capacity to profile bacterial communities. Tremblay et al.^11^ also found quantitative differences between V4, V6V8, V7V8 regions in environmental samples, not affecting the detected structural pattern. In our study, in saliva samples, two oral types were still differentiated irrespective of the target region (Fig. 5a), and no clustering according to 16S rRNA target domain could be observed (Fig. 5b). Thus, in saliva samples, all domains showed a similar capacity to profile microbial communities and segregate oral types accordingly.

In accordance with what has been previously exposed, multivariant PERMANOVA test revealed statistical differences in soil development or oral types and target regions at the genus level (Table 2). All soil samples were statistically different from each other in PERMANOVA pairwise tests, which had been previously reported by Sánchez-Marañón et al.^26^, and so were oral types (Supplementary Table S2).

All domains differed in soil samples while only V4V5 differed from V1V3 and V6V8 in saliva samples (Supplementary Table S2). The analysis of the differences between 16S rRNA regions in each soil type showed a higher influence of 16S rRNA domains in developed soils S7 and S8 as statistical differences between V1V3 and V3V4 regions were detected only in those soils (Supplementary Table S3). The analysis of the differences between soil types in each 16S rRNA region revealed that differences between the lowest developed soils S1–S2 were only detected by V4V5 region, suggesting a higher sensitivity of this region for soils with low development (Supplementary Table S4). On the contrary, in saliva samples, the differences between target regions were not affected by the oral type, and neither were differences between oral types affected by the target region (Supplementary Table S5), suggesting that 16S rRNA regions had a more limited influence on the outcome of microbiome studies regarding saliva samples.

Having evaluated the overall effect of the 16S rRNA region on the characterization of microbiome structure, a specific assessment of microbial genera by the different domains was still needed. A mounting interest in the establishment of microbial signatures and microbial biomarkers and their relationship with several illnesses and environmental processes is awakening in the scientific community given their great potential for diagnosis, prognosis and treatment response in both types of samples^37^. The detection of biomarkers related to soil development^26^, such as *Terrimonas*, *Gemmataceae_uncultured*, *Armatimonadetes_uncultured_ge* and *Solirubrobacter* varied considerably between 16S rRNA regions (Fig. 2, Supplementary file 2, Supplementary Fig. S9). In the case of saliva samples, *Campylobacter, Solobacterium, Erysipelotrichia, Fusobacterium* and *Leptotrichia* genera were also differently detected by 16S rRNA target regions (Fig. 3). Several studies have reported the importance of those genera in disorders such as nasopharyngeal carcinoma, esophageal adenocarcinoma or HIV^36,38,39^, and therefore, a thorough selection of primer sets should be made when designing studies in those areas.

Lastly, in terms of bacterial diversity, V1V3 domain showed the highest values for the majority of alpha diversity parameters in soil and saliva samples and the lowest bacterial coverage (Fig. 1, Supplementary Fig. S2). These results are in accordance with those obtained by Whon et al., attributing this increased alpha diversity to the longer amplicon length in the V1V3 region^3^. Increased amplicon length implies higher polymerase-associated errors, as well as those derived from base-calling and read merging. The percentaje of low-quality sequences removed along the bioinformatic pipeline showed that V1V3 was the region with the greatest percentage of removed sequences, although only a 5% difference was noticed compared to V6V8 in soil and saliva samples (Table 1). The percentage of removed sequences varied according to the sample type (soil or saliva); namely, V3V4 region suffered a greater reduction in the number of sequences in soils compared to saliva samples (Table 1). Nevertheless, amplicon length is not always correlated with increased alpha diversity, since the longest region (V1V3) showed lower values than V3V4 for Chao1 index in soil samples (Fig. 1). Similarly, V3V4 region showed lower values for the number of observed OTUs and Chao1 index than V6V8 in saliva samples (Fig. 1); higher values for InvSimpson and Pielou were noticed for V4V5 region compared to V6V8 in saliva samples. This could be explained by well-documented differences in the detection of microbial taxa by the different primer sets^5^.

In light of the above, and considering that the use of different target regions might skew the detection of specific bacteria, especially in soil samples, an assessment of which region yielded the most reliable data was still needed. For that purpose, mock community B (HM-782D), enshrined within the Human Microbiome Project and considerably used as a NGS control^8,40^, was sequenced using V1V3, V3V4, V4V5 and V6V8 primer sets. The obtained microbial profiles were compared against mock community theoretical composition, bearing the V1V3 region the closest resemblance at the genus level, followed by V3V4 domain. V1V3 domain was the region that detected the highest number of genera in soil and saliva samples and the only one detecting the template DNA from all members in mock community B (Supplementary Fig. S11). Additionally, it presents the longest amplicon length providing an increased taxonomic accuracy. Similar results were obtained by Albertsen et al.^9^, who used shotgun metagenomics as a PCR-free sequencing control instead, while Whon et al.^3^ found the highest correlation for V3V4 region. All amplified regions were more similar to each other than to the expected profile (Table 3), which could be attributed to the fact that they underwent the same amplifying and sequencing procedure and bioinformatic pipeline. PCR amplifying protocols, and in particular, factors such as the amount of template DNA, number of cycles or annealing temperature might affect profiling of microbial communities^9,41^. Additionally, following similar bioinformatic pipelines has been shown to be of great importance when comparing results, even between different sequencing platforms and primer sets^41,42^. These results emphasize the importance of a consensus regarding NGS protocols in order to facilitate comparability between studies.

Environmental samples included in this analysis are Mediterranean calcarean soils, and given the great variability and ecological complexity associated with soil samples, further studies involving other types of soils might be necessary to corroborate the obtained results. On the other hand, saliva samples are characterized by low beta diversity, thus suggesting these results might be applicable for a wide variety of salivary microbiome studies. There is no denying the fact that, in all likelihood, primer sets will not completely meet all project demands, and therefore, this study aims to provide guidance about key bacterial taxa that might be influenced by the choice of different 16S rRNA target regions in soil and saliva microbiome studies. Longer amplicon length (V1V3, V3V4) implies higher taxonomic accuracy, while overestimation of alpha diversity is affected by additional factors such as bias in the detection of specific taxa by the different primer sets. Sequencing errors that could still remain after bioinformatic processing would not affect taxonomic analysis since classifiers use cutoffs of 80% by default. Thorough designing and careful selection of primers depending on the objective of the research is essential in amplicon sequencing analysis to avoid skews in the obtention of results.

## Methods

### Sample collection and DNA extraction

Soil samples were collected as previously described^26^. Briefly, eight soils selected along a pedogenic gradient (S1–S8 in increasing order of development) at the local scale in a Mediterranean calcareous mountain were sampled in four spatial replicates making a total of 32 soil samples. Saliva samples belonging to 11 healthy donors (6 females and 5 males, mean age: 37.09 ± 12.87) were collected in the morning, immediately after waking up, by oral rinsing with sterile 1 × Phosphate buffer saline. Samples were centrifuged (6,000 rpm) for 10 min upon arrival to the lab and supernatants were carefully discarded. Pellets were immediately frozen at – 80 °C until DNA isolation procedures were performed. Lastly, DNA from Microbial Mock Community B (Even, Low Concentration), v5.1L, for 16S rRNA Gene Sequencing (HM-782D) was used as an internal sequencing control and obtained through BEI Resources, NIAID, NIH as part of the Human Microbiome Project.

DNA isolation from soil samples was as previously described^26^, and so was DNA isolation from saliva samples^43^, in this case with some modifications. Briefly, pellets were thawed and dissolved in 200 µL lysis buffer 2× (3% w/v sodium dodecyl sulphate in 50 mM tris, 5 mM EDTA, pH 8.0, 10 µg/ml RNase A) at 68 °C for 1 h, following from this point the subsequent procedure. Negative controls were included in all extraction batches and were subsequently amplified to assess the presence of contaminants. DNA quality and amount were determined using a spectrophotometer (NanoDrop 2000 UV–Vis ThermoFisher Scientific, Waltham, MA, USA).

Reproducibility was assessed in soil samples owing to its greater complexity and variability. Technical validation included DNA isolation, PCR amplification and sequencing procedures using V1V3 primer conditions, since this region was the most likely to introduce bias due to its increased amplicon length.

### High-throughput sequencing and bioinformatics analysis

PCR amplification products of the V1V3, V3V4, V4V5 and V6V8 variable regions of the 16S rRNA gene were obtained in all sample types (soils, saliva and mock community) using fusion universal primers: V1V3 (F: 5′-Illumina adaptor 1 + barcode + Illumina adaptor 2 + AGAGTTTGATCMTGGCTCAG-3′, R: 5′-Illumina adaptor 1 + barcode + Illumina adaptor 2 + TTACCGCGGCKGCTGGCACG-3′), V3V4 (F: 5′-Illumina adaptor 1 + barcode + Illumina adaptor 2 + CCTACGGGNGGCWGCAG-3′ , R: 5′-Illumina adaptor 1 + barcode + Illumina adaptor 2 + GACTACHVGGGTATCTAATCC-3′), V4V5 (F: 5′-Illumina adaptor 1 + barcode + Illumina adaptor 2 + GTGYCAGCMGCCGCGGTAA-3′, R: 5′-Illumina adaptor 1 + barcode + Illumina adaptor 2 + CCGYCAATTYMTTTRAGTTT-3′), V6V8 (F: 5′-Illumina adaptor 1 + barcode + Illumina adaptor 2 + AATTGACGGGGRCCCGC-3′, R: 5′-Illumina adaptor 1 + barcode + Illumina adaptor 2 + ACGGGCRGTGWGTRCAA-3′). V4V5 primers have been previously described by Parada et al.^34^ and Walters et al.^44^. V1V3, V3V4 and V6V8 primers have also been described by Klindworth et al.^5^. In our study V1V3 reverse primer and V6V8 primers were slightly modified to enhance bacterial coverage^4^. Briefly, V1V3 reverse primer was degenerated for guanine or thymine in position eleven,V6V8 forward primer is one bp shorter in 5′ end than its counterpart described by Klindworth et al.^5^, while V6V8 reverse primer was degenerated for adenine or guanine in position seven. Similarly, negative controls were included in each PCR batch to assess the presence of contaminants in the amplification step. Both extraction and PCR negative controls were sequenced using the whole PCR product. PCR settings included a total of 25 cycles to reduce PCR artifacts^45^ in a single PCR using complete fusion universal primers and annealing temperature of 55 °C. Amplicon multiplexing and sequencing was carried out with a dual indexing tag-tailed design using 8nt indexes from the Nextera XT Index Kit v2 (Illumina, San Diego, CA, USA). Paired-end sequencing of 16S rRNA amplicon libraries was performed using the Illumina MiSeq instrument with v3 kit chemistry (300 + 300). Demultiplexing was performed by Illumina BaseSpace software with default settings. Bioinformatics analysis and quality-filtering were carried out using the software Mothur v1.43.0 (University of Michigan Medical School, Ann Arbor, MI, USA)^46^, following the standard Miseq SOP. Mothur standard pipeline consists of an initial step combining the forward and reverse read with default parameters, followed by a first quality-filtering aiming to remove sequences with ambiguous bases, length lower than 299 bp or over 525 bp, or more than 9 homopolymers. The subsequent processing proceeded with the allocation of unique sequences and the creation of a count table. Alignment against Silva v132 database was next carried out with default parameters, continuing with a second quality-filtering to eliminate poorly aligned sequences. After a reduction in the number of columns in the alignment, unique sequences are again allocated and pre-clustered considering up to 4 sequencing errors. Lastly, chimeric reads were identified and excluded using Chimera UCHIME. Diversity was examined by opticlust method defining operational taxonomic units (OTUs) at 3% dissimilarity; coverage, number of observed OTUs, richness index Chao1, specific-diversity indexes (InvSimpson, Shannon) and evenness index Pielou were calculated as alpha diversity variables. Redundant, non-chimera FASTA sequences were taxonomically classified using Silva v132 as a reference and the bayesian classifier described by Wang et al.^47^. Abundance was expressed as a percentage with respect to the total number of sequences in each sample. Genera with total abundance higher than 0.05% were considered for statistical analysis.

### Statistical analysis

After checking the absence of normality in the variables with the Shapiro–Wilk test using SPSS v.20.0 (SPSS Inc., Chicago, IL, USA)., permutational analysis of variance (PERMANOVA) test was performed in all samples. Univariant PERMANOVA test was carried out using R software (R Foundation for Statistical Computing, Vienna, 2012) and euclidean distances. Multivariant PERMANOVA test and pairwise test using Bray–Curtis similarity was implemented on PRIMERe Permanova + (PRIMER-E Ltd, Plymouth, UK), as well as resemblance matrixes. Pre-treatment analysis (square roots) were performed prior to the implementation of PERMANOVA and Resemblance Matrix tests due to considerable differences in bacterial abundance. In all PERMANOVA analyses, 9999 permutations of the residuals under the full model were used. A p value of 0.05 was considered to determine statistical differences unless otherwise indicated.

Principal coordinate analysis (PCoA) based on Bray–Curtis distances were implemented in PRIMERe Permanova+, while principal component analysis (PCA) was performed using Statgraphics Centurion XVII (Statpoint Technologies, Inc., Warrenton, VA, USA) and R software (R Foundation for Statistical Computing, Vienna, 2012).

Lastly, Linear discriminant analysis Effect Size (LEfSe) was carried out using Python 3.7.6, considering a p value of 0.05 and LDA of 2^48^. To facilitate graphical representation and reduce the number of shown features in soil samples, p value and LDA were adjusted to 0.01 and 3.5 respectively.

### Ethics statement

All saliva samples were collected from volunteers and written informed consent was obtained from all participants according to the ethical guidelines approved by the Regional Health Authority (Junta de Andalucía, PEIBA: 0699-N-20).

## Supplementary information

Supplementary Dataset 1.

Supplementary Dataset 2.

Supplementary Dataset 3.

Supplementary Dataset 4.

Supplementary Dataset 5.

Supplementary Information 1.

Supplementary Information 2.

Supplementary Dataset 6.

Supplementary Information 3.

## Data Availability

All datasets supporting the conclusions of this article are available in the Sequence Read Archive (SRA) of the National Centre for Biotechnology Information (NCBI) under the bioproject number PRJNA612815 (SRA numbers SRR11321553-SRR11321728). For further information on SRA and Biosample accession numbers, refer to Supplementary File 8. Authors can confirm that all relevant data are included in the article and/or its supplementary information files.
