# Supplementary material for: Influence of 16S rRNA target region on the outcome of microbiome studies in soil and saliva samples
Source: Sci Rep. 2020 Aug 12;10:13637. doi: 10.1038/s41598-020-70141-8 (PMC7423937; doi:10.1038/s41598-020-70141-8)
Supplement: Supplementary file 9 — Supplementary Information 3. [file 41598_2020_70141_MOESM9_ESM.pdf]

Influence of 16S rRNA target region on the outcome of microbiome studies in soil and saliva samples

Ana Soriano-Lerma[1,2], Virginia Pérez-Carrasco[2,3], Manuel Sánchez-Marañón[4], Matilde Ortiz-González[2,5], Victoria Sánchez-Martín[2,3], Juan Gijón[6], José María Navarro-Mari[3], José Antonio García-Salcedo[2,3] *, Miguel Soriano[2,5] *

[1]Department of Physiology (Faculty of Pharmacy, Campus Universitario de Cartuja), Institute of Nutrition and Food Technology "José Mataix", University of Granada, E-18071 Granada, Spain. [2]GENYO. Centre for Genomics and Oncological Research: Pfizer / University of Granada / Andalusian Regional Government, PTS Granada, E-18016, Granada, Spain. [3]Microbiology Unit, Biosanitary Research Institute ibs.GRANADA, University Hospital Virgen de las Nieves, E-18014, Granada, Spain. [4]Department of Soil Science and Chemical Agriculture, University of Granada, 18071 Granada, Spain. [5]Center for Intensive Mediterranean Agrosystems and Agri-food Biotechnology (CIAIMBITAL), University of Almeria, E-04001, Almería, Spain. [6]Department of Periodontics, School of Dentistry, University of Granada, Granada, Spain.

SUPPLEMENTARY INFORMATION

Supplementary Figures S1-S11

Supplementary Tables S1-S5

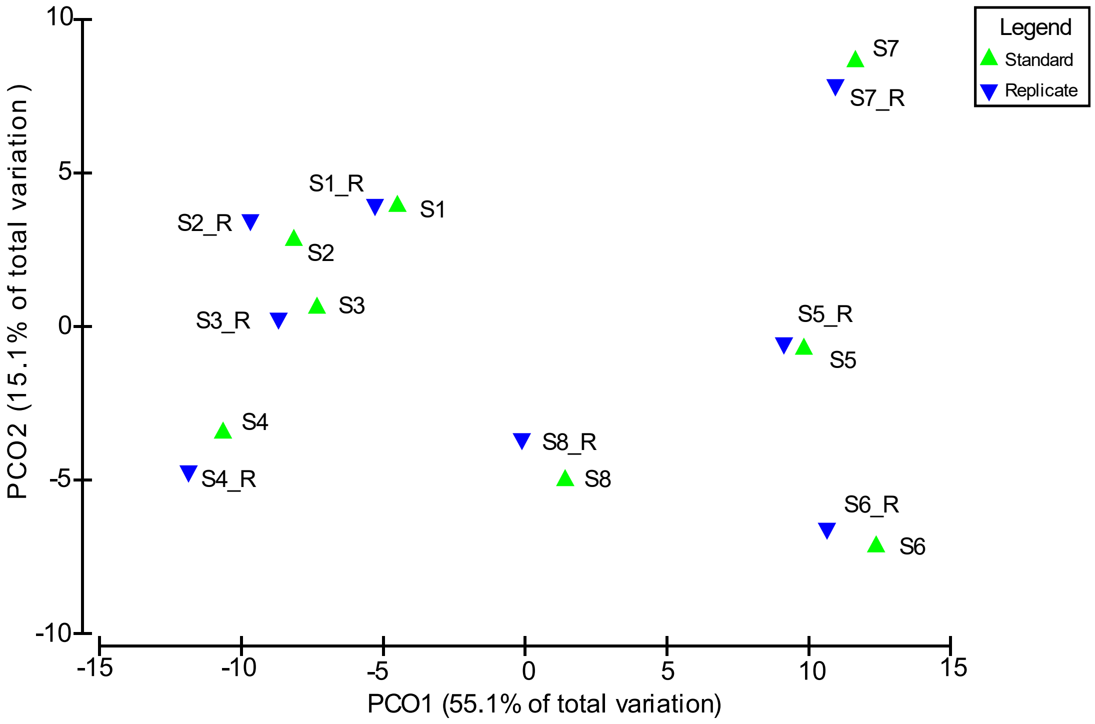

Supplementary Figure S1. Principal coordinate analysis (PCoA) based on Bray-Curtis distances. Plots for the bacterial genera with a relative abundance higher than 0.05% in technical replicates from 8 different types of soil sequenced under V1V3 primer conditions. Samples are represented by coloured symbols according to the legend; samples in green were included in subsequent analysis, while samples in blue represent its respective technical replicate (DNA isolation and PCR amplification). PRIMER e Permanova + (PRIMER-E Ltd, Plymouth, UK) was used in the implementation of the statistical analysis.

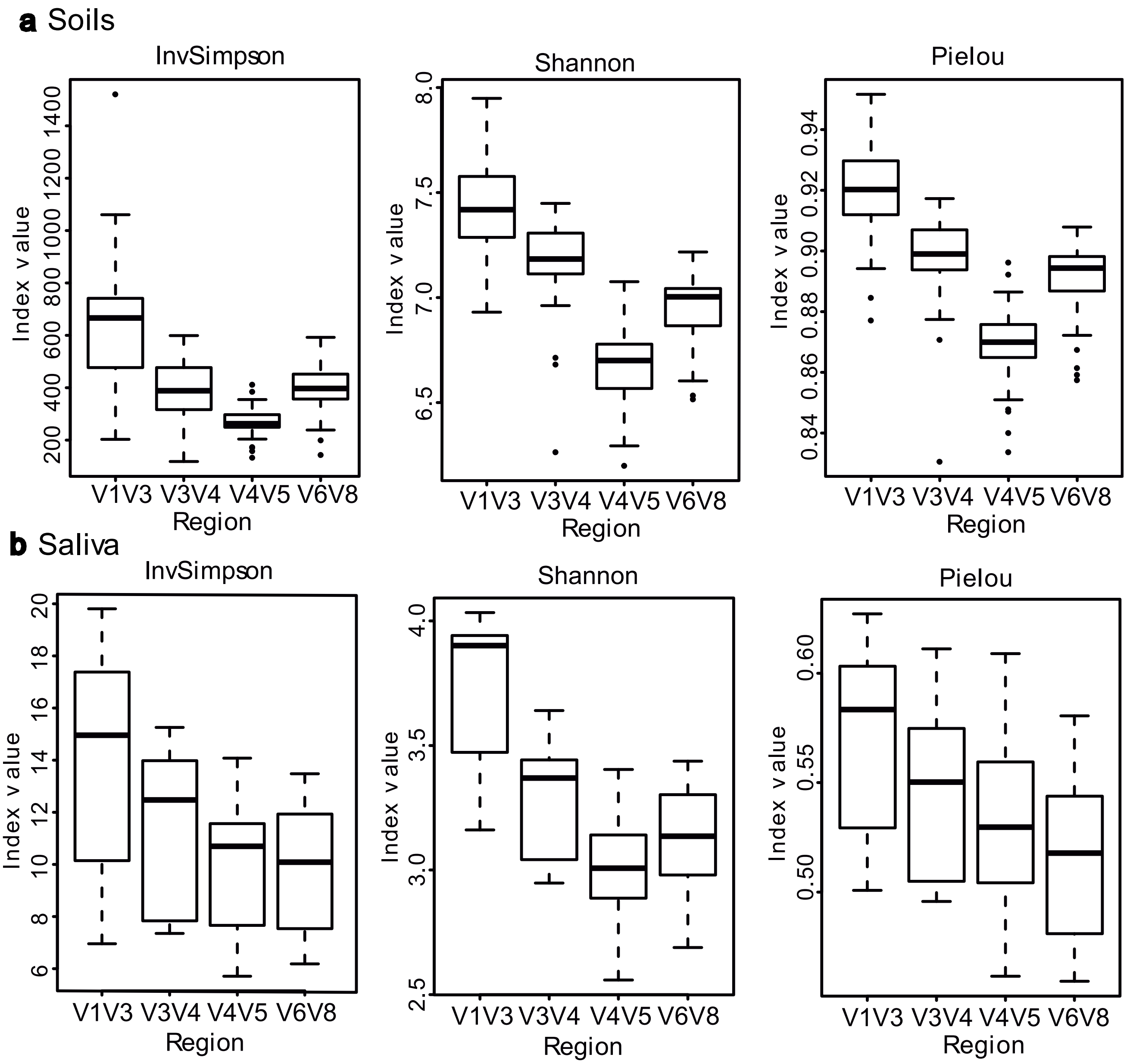

Supplementary Figure S2. Alpha diversity variables in each 16S rRNA region considering soil (a) and saliva samples (b). Specific diversity indexes InvSimpson and Shannon, and evenness index Pielou were calculated defining OTU at 3% of dissimilarity by opticlust method (Mothur v1.43.0 software, University of Michigan Medical School, Ann Arbor, MI, USA). (a) Specific diversity indexes InvSimpson and Shannon, and evenness index Pielou in each 16S rRNA region considering all soil samples (n=32). (b) Specific diversity indexes InvSimpson and Shannon, and evenness index Pielou in each 16S rRNA region considering all saliva samples (n=11).

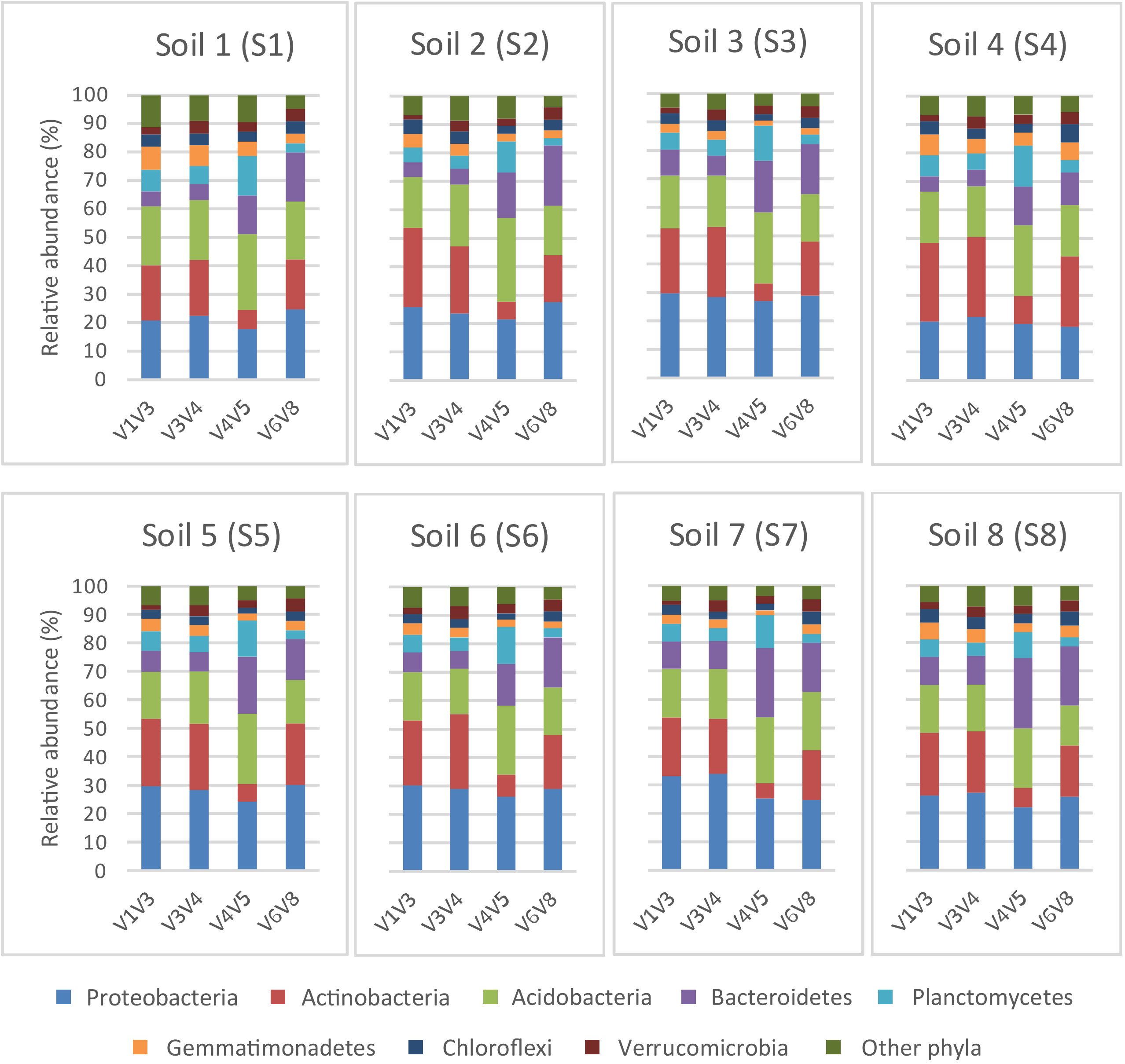

Supplementary Figure S3. Taxonomic distribution considering relative abundances at the phylum level in soil samples. The most abundant phyla and each 16S rRNA region were included. Each soil type (S1-S8) includes mean values for four spatial replicates (n=4)

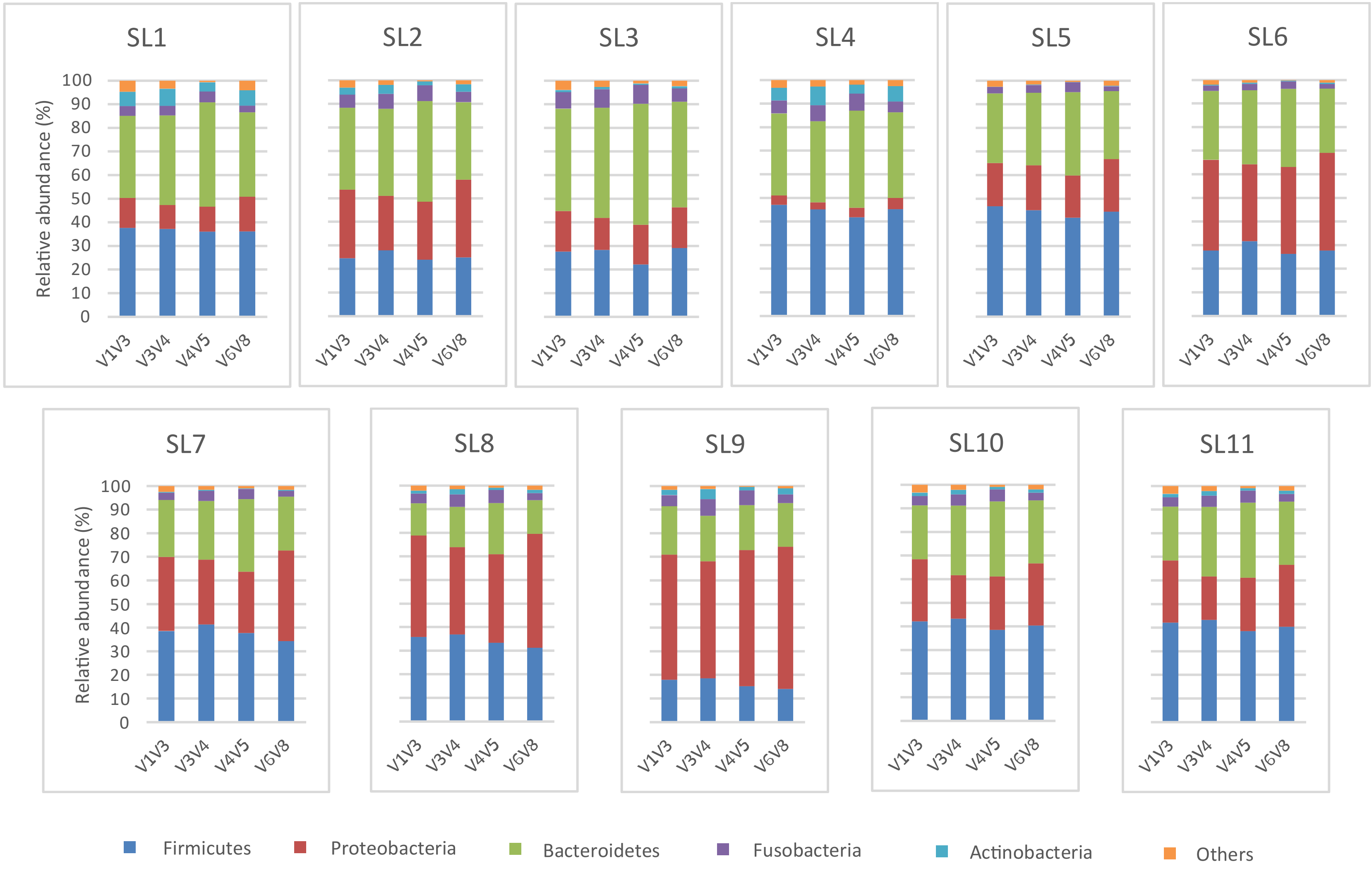

Supplementary Figure S4. Taxonomic distribution considering relative abundances at the phylum level in saliva samples. The most abundant phyla and each 16S rRNA region were included. Each sample represents one replicate.

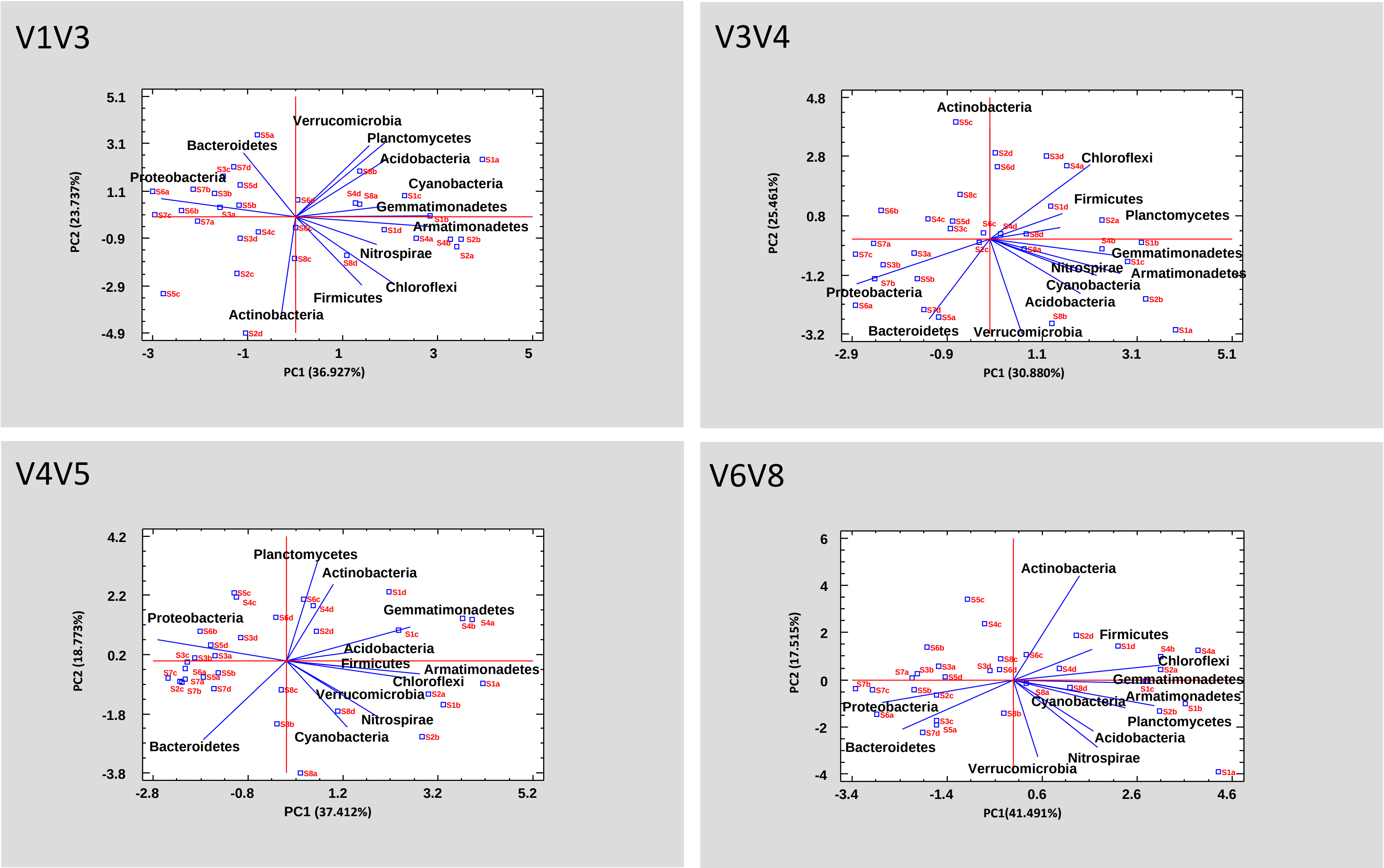

Supplementary Figure S5. Principal component analysis (PCA). Biplots for the relative abundance of bacterial phyla in soil samples considering the most abundant and differently distributed phyla. Samples are represented by dots and phyla are represented by lines. Red lines represent the coordinates axis. S1-S8 represents the type of soil, while letters a, b, c and d represent the four spatial replicates for each type of soil. Statgraphics Centurion XVII (Statpoint Technologies, Inc., Warrenton, VA, USA) was used in the implementation of the statistical analysis.

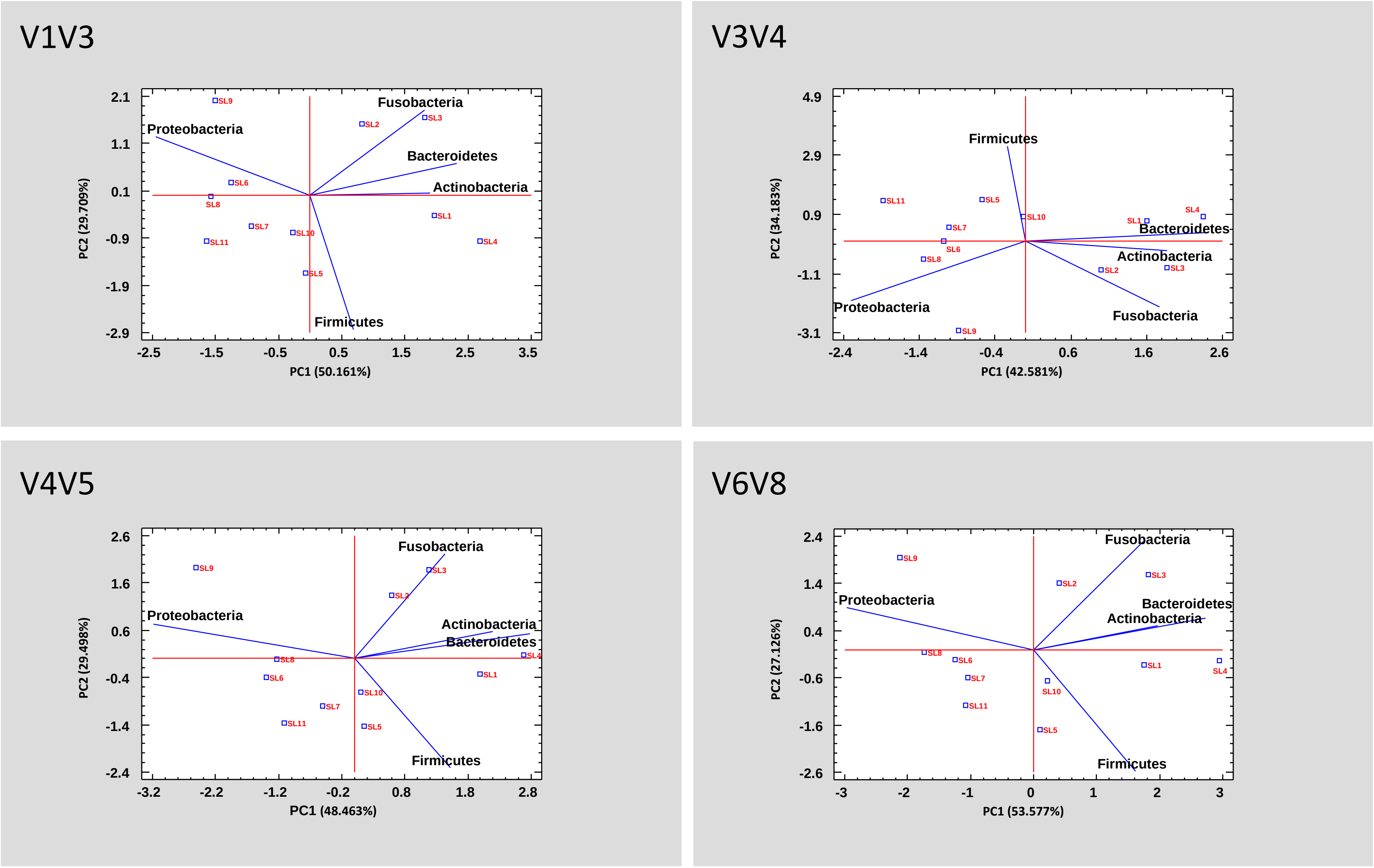

Supplementary Figure S6. Principal component analysis (PCA). Biplots for the relative abundance of bacterial phyla in saliva samples considering the most abundant phyla. Samples are represented by dots and phyla are represented by lines. Red lines represent the coordinates axis. SL1-SL11 represents each saliva sample. Statgraphics Centurion XVII (Statpoint Technologies, Inc., Warrenton, VA, USA) was used in the implementation of the statistical analysis.

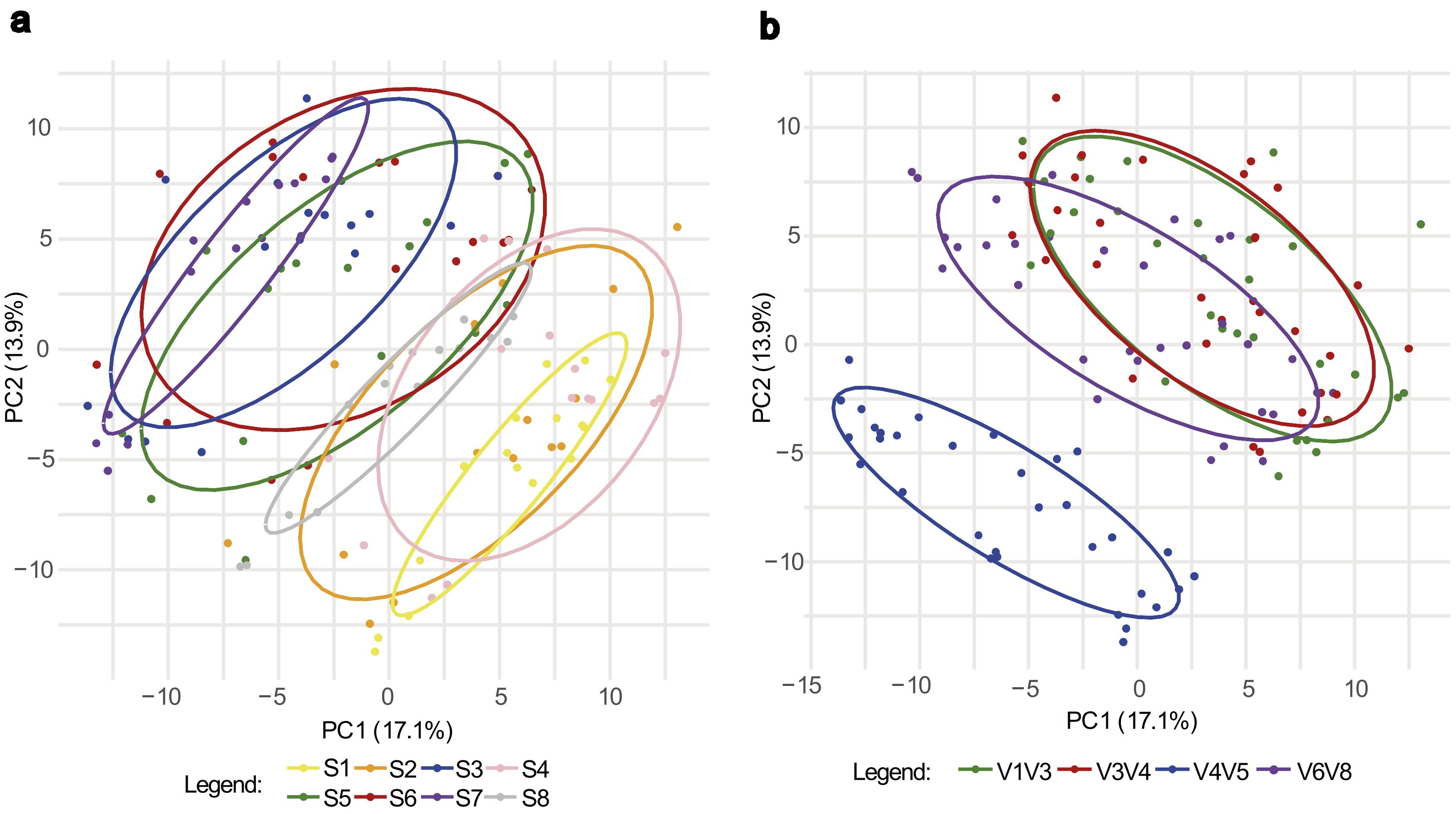

Supplementary Figure S7. Principal component analysis (PCA). Plots for the bacterial genera with a relative abundance higher than 0.05% in soil samples. Samples are represented by dots and colour elypses have been added to represent clustering of soils along a pedogenic gradient (a) or around 16S rRNA regions (b). (a) Different types of soil with their spatial respective replicates are considered regardless of 16S rRNA region (n=128). (b) Different 16S rRNA regions are considered regardless of the type of soil (n=128). R software (R Foundation for Statistical Computing, Vienna, 2012) was used in the implementation of the statistical analysis.

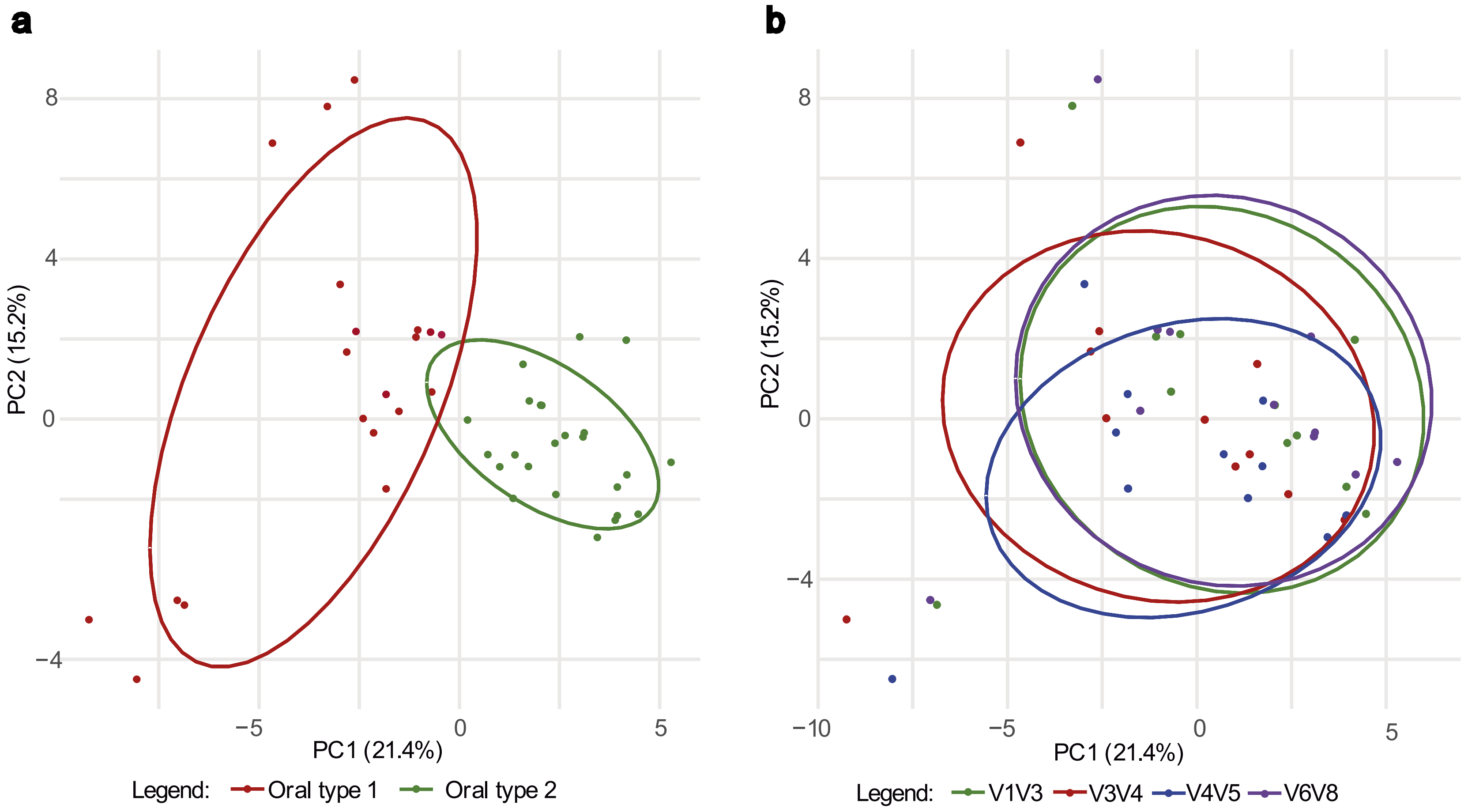

Supplementary Figure S8. Principal component analysis (PCA). Plots for the bacterial genera with a relative abundance higher than 0.05% in saliva samples. Samples are represented by dots and colour elypses have been added to represent the clustering around oral types (a) or around 16S rRNA regions (b). (a) Different oral types are considered regardless of 16S rRNA region (n=44). (b) Different 16S rRNA regions are considered regardless of the respective saliva sample (n=44). R software (R Foundation for Statistical Computing, Vienna, 2012) was used in the implementation of the statistical analysis.

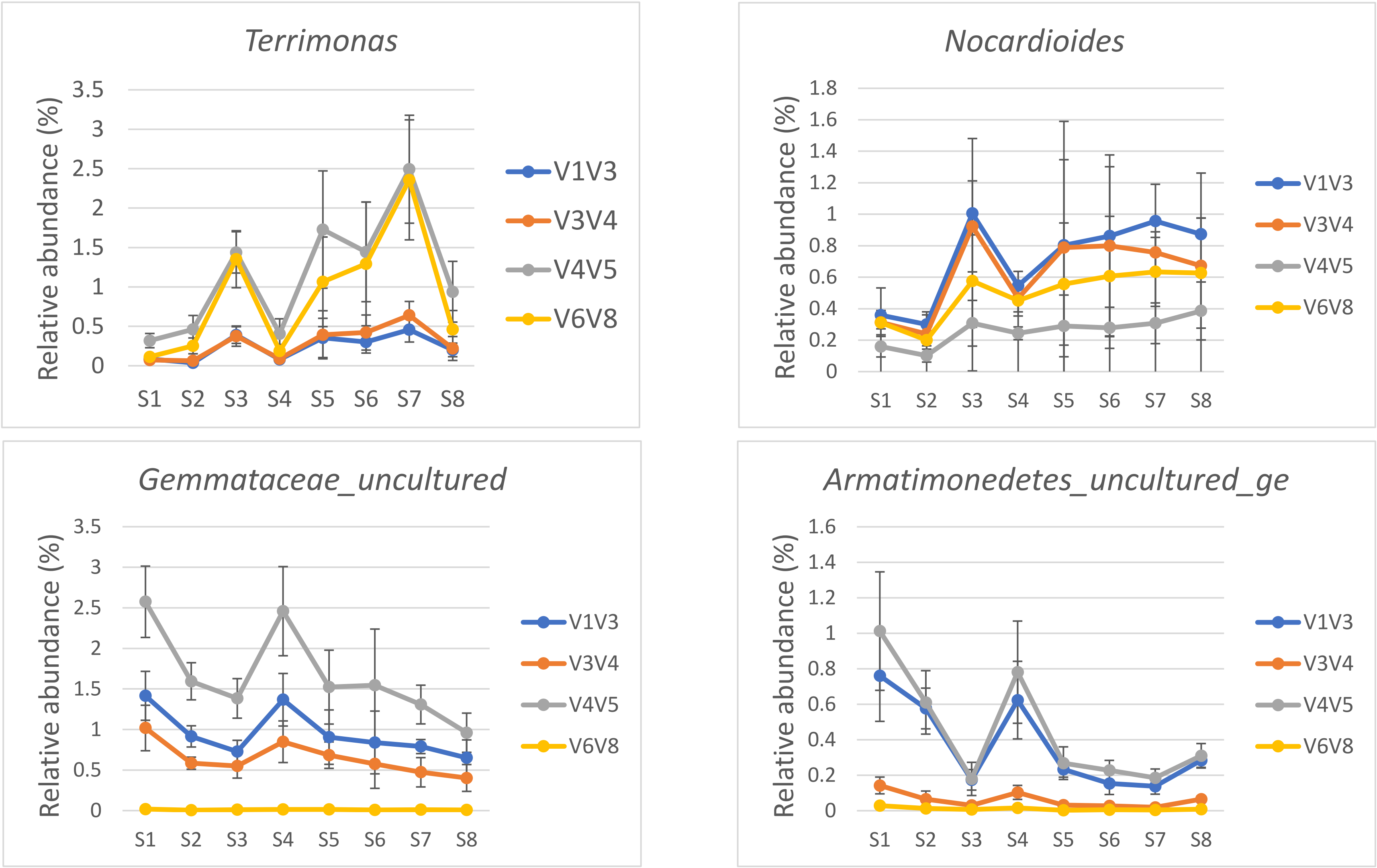

Supplementary Figure S9. Bacteria associated with soil development showing statistical differences between 16S rRNA regions in LEfSe analysis. Colour lines represents the variation in mean relative abundance along soils S1-S8 in each 16S rRNA region (n=4). Standard deviations between all replicates in each soil and region are shown as error bars.

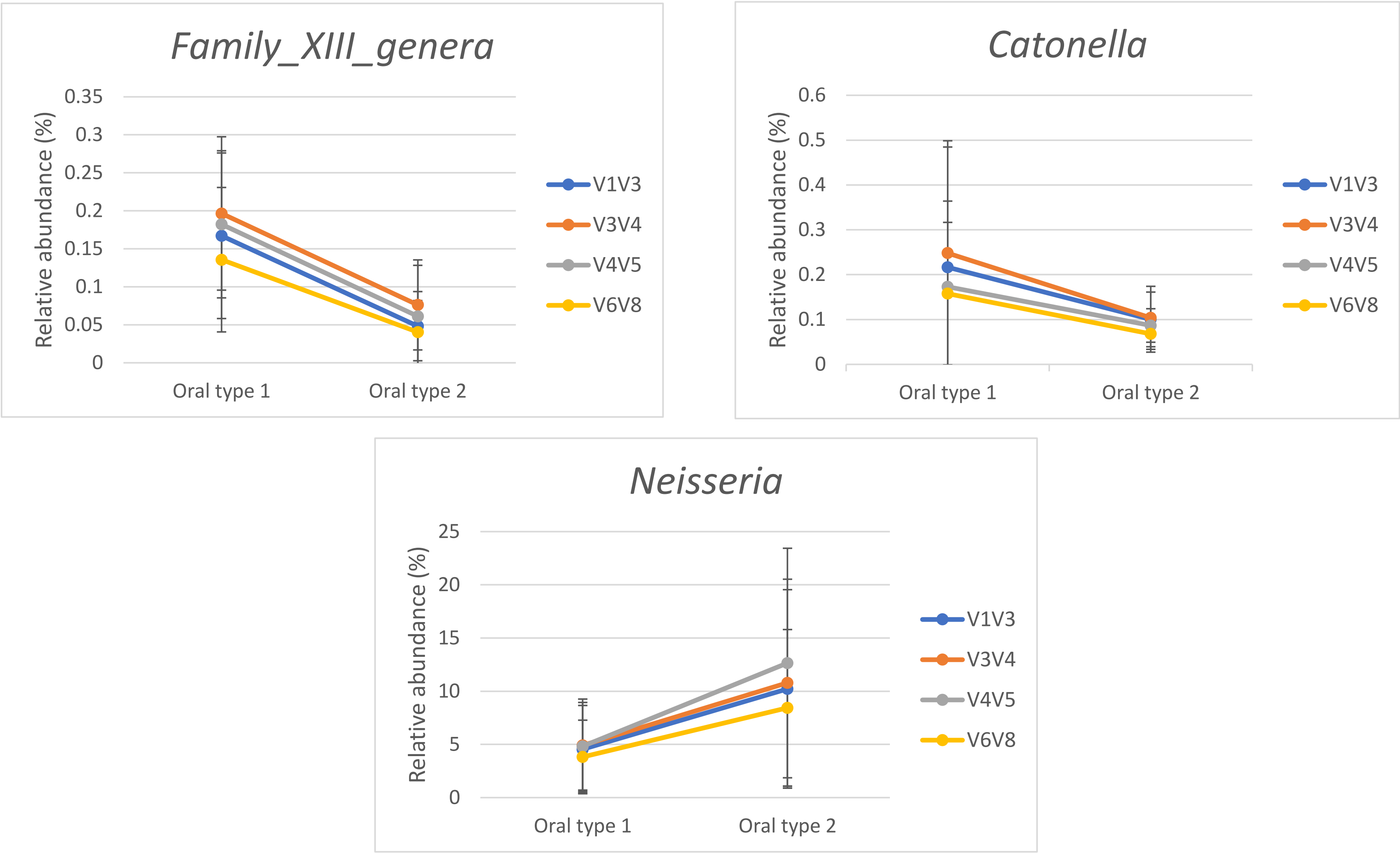

Supplementary Figure S10. Oral type-related genera showing no statistical differences between 16S rRNA regions in LEfSe analysis. Colour lines represents the variation in mean relative abundance between oral type 1 and 2 in each 16S rRNA region (n=5 for oral type 1; n=6 for oral type 2). Standard deviations in each oral type and region are shown as error bars.

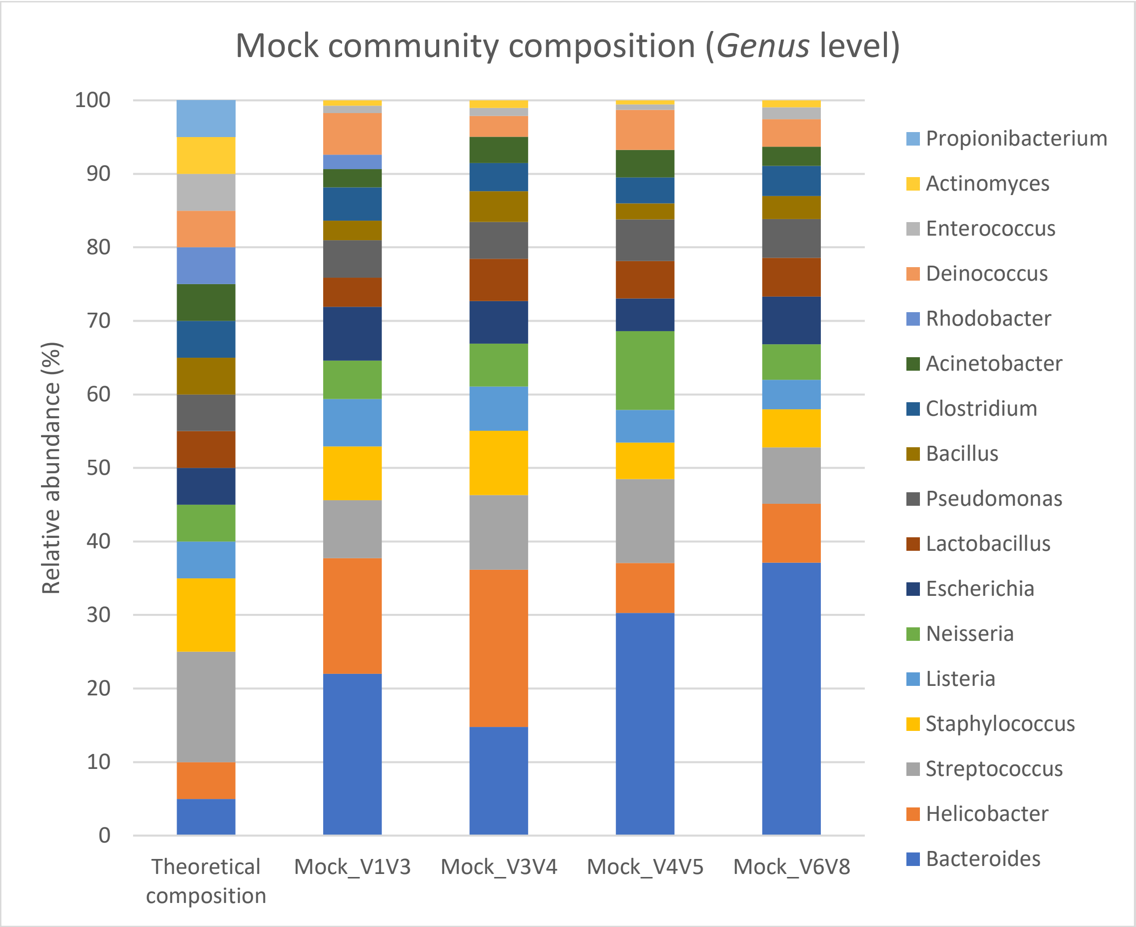

Supplementary Figure S11. Taxonomic distribution at the genus level considering theoretical composition in mock community B and relative abundances in mock community B amplified with V1V3, V3V4, V4V5 and V6V8 regions.

Supplementary Table S1. 20 main contributing variables for principal component (PC) 1 and 2, ordered according to their positive or negative correlation and their loading (those contributing to both PCs were ordered according to their values in PC1). * and ^ indicates the selected variables; Soils: ^ for variables with negative (or near zero) values in PC1 and positive (or near zero) loadings in PC2; * for variables with positive (or near zero) values in PC1 and negative (or near zero) values in PC2; Saliva: ^ for variables with negative loadings (high values or those included between -0.5 and 0) in PC1, along with negative or positive high values in PC2; * for variables with positive high values in PC1 and negative values ranging from -0.5 and 0 in PC2. R software (R Foundation for Statistical Computing, Vienna, 2012) was used in the implementation of the statistical analysis.

SOIL SAMPLES				SALIVA SAMPLES			
	PC1	PC2	Contribute to PC		PC1	PC2	Contribute to PC
Gaiellales_unclassified*	0.769	-0.178	1	Haemophilus*	0.615	0.083	1
S085_ge	0.746	0.190	1	Pasteurellaceae_unclassified*	0.576	-0.108	1
Gaiellales_uncultured_ge*	0.742	-0.060	1	Aggregatibacter*	0.533	-0.161	1
Solirubrobacterales_unclassified	0.705	0.316	1	Granulicatella*	0.497	-0.095	1
Gaiella	0.704	0.192	1	Neisseria*	0.496	-0.110	1
Acidobacteria_subgroup_5_ge	-0.716	-0.175	1	Oribacterium^	-0.570	0.580	1 and 2
KF-JG30-B3_ge^	-0.717	0.191	1	Treponema_2^	-0.612	-0.610	1 and 2
Chryseolinea^	-0.719	0.066	1	Rothia	-0.616	-0.178	1
Paludibaculum^	-0.728	0.217	1	Family_XIII_ge^	-0.620	0.665	1 and 2
Ohtaekwangia	-0.730	-0.249	1	Prevotella_7^	-0.625	0.538	1
Sphingobacteriales_unclassified	-0.732	-0.120	1	Selenomonas_3	-0.677	0.169	1 and 2
env.OPS_17_ge	-0.740	-0.271	1	Catonella^	-0.682	-0.495	1 and 2
Saprospiraceae_uncultured^	-0.748	0.164	1	Campylobacter^	-0.682	0.408	1 and 2
Flavobacterium^	-0.751	0.080	1	Lachnoanaerobaculum^	-0.687	-0.376	1
Micropepsaceae_uncultured^	-0.755	0.288	1	F0058^	-0.706	-0.561	1 and 2
AKYH767_ge^	-0.768	0.187	1	Dialister^	-0.727	-0.550	1 and 2
Lineage_IIa_ge^	-0.782	0.196	1	Prevotella	-0.745	-0.339	1
Ferruginibacter	-0.821	-0.044	1	Peptostreptococcus^	-0.756	-0.477	1 and 2
NS9_marine_group_ge^	-0.825	0.101	1	Tannerella^	-0.768	-0.481	1 and 2
Terrimonas^	-0.830	-0.003	1	Actinomyces	-0.850	0.219	1
Iamia^	0.007	0.775	2	Atopobium^	-0.399	0.840	2
Haliangium^	-0.263	0.707	2	Saccharimonadaceae_ge^	-0.330	0.805	2
IMCC26256_ge	0.354	0.678	2	Candidatus_Saccharimonas^	-0.289	0.765	2
Microtrichales_unclassified	0.310	0.674	2	Megasphaera^	-0.463	0.713	2
Rhizobiales_unclassified	0.104	0.673	2	Solobacterium^	-0.377	0.709	2
Xanthobacteraceae_uncultured	0.038	0.666	2	Firmicutes_unclassified	0.014	0.565	2
Micromonosporaceae_unclassified	0.318	0.663	2	Prevotellaceae_unclassified^	-0.282	0.425	2
Nocardioides^	-0.040	0.654	2	Prevotella_2^	-0.239	-0.438	2
Alphaproteobacteria_unclassified^	-0.114	0.649	2	Parvimonas^	-0.446	-0.533	2
Solirubrobacteraceae_uncultured^	-0.133	0.647	2	Streptobacillus^	-0.493	-0.658	2
Solirubrobacter	0.183	0.639	2				
KD4-96_ge	0.336	0.639	2				
Gemmataceae_uncultured*	-0.033	-0.626	2				

Armatimonadetes_uncultured_ge*	0.189	-0.630	2
Rokubacteriales_ge*	0.220	-0.637	2
Flavisolibacter	-0.331	-0.641	2
Gemmataceae_unclassified	-0.257	-0.642	2
Acidobacteria_subgroup_6_unclassified	-0.533	-0.657	2
FBP_ge*	0.077	-0.667	2
WD2101_soil_group_ge*	0.166	-0.696	2

Supplementary Table S2. Multivariant PERMANOVA pairwise test. Determination of statistical differences in taxonomic variables within each level of each factor in soil (n=128) and saliva samples (n=44). PRIMER e Permanova + (PRIMER-E Ltd, Plymouth, UK) was used in the implementation of the statistical analysis.

Soil samples: region

Groups	t	P(perm)	Sig
V1V3,V3V4	3.422	0.0001	*
V1V3,V4V5	7.21	0.0001	*
V1V3,V6V8	6.644	0.0001	*
V3V4,V4V5	7.194	0.0001	*
V3V4,V6V8	6.169	0.0001	*
V4V5,V6V8	8.445	0.0001	*

Saliva samples: region

Groups	t	P(perm)	Sig
V1V3,V3V4	1.142	0.2484	n.s.
V1V3,V4V5	1.557	0.0233	*
V1V3,V6V8	1.176	0.2203	n.s.
V3V4,V4V5	1.101	0.3036	n.s.
V3V4,V6V8	1.24	0.1535	n.s.
V4V5,V6V8	1.623	0.0165	*

Soil samples: soil

Groups	t	P(perm)	Sig
S1,S2	1.887	0.0153	*
S1,S3	2.005	0.0054	*
S1,S4	2.667	0.0002	*
S1,S5	2.94	0.0002	*
S1,S6	4.044	0.0001	*
S1,S7	3.394	0.0001	*
S1,S8	3.91	0.0001	*
S2,S3	2.043	0.0047	*
S2,S4	2.959	0.0001	*
S2,S5	3.754	0.0001	*
S2,S6	4.671	0.0001	*
S2,S7	3.822	0.0001	*
S2,S8	4.604	0.0001	*
S3,S4	2.724	0.0001	*
S3,S5	4.005	0.0001	*
S3,S6	5.618	0.0001	*
S3,S7	4.779	0.0001	*
S3,S8	4.822	0.0001	*
S4,S5	4.987	0.0001	*
S4,S6	6.769	0.0001	*
S4,S7	5.901	0.0001	*
S4,S8	5.616	0.0001	*
S5,S6	2.65	0.0002	*
S5,S7	2.738	0.0005	*
S5,S8	3.832	0.0001	*

S6,S7	2.472	0.0002	*
S6,S8	4.505	0.0001	*
S7,S8	4.336	0.0001	*

Saliva samples: oral type

Groups	t	P(perm)	Sig
O1,O2	3.284	0.0001	*

Supplementary Table S3. Multivariant PERMANOVA pairwise test. Determination of statistical differences between 16S rRNA regions in each type of soil (n=32). PRIMER e Permanova + (PRIMER-E Ltd, Plymouth, UK) was used in the implementation of the statistical analysis.

Soil samples: region*soil; factor: region

Within level 'S1' of factor 'Soil'				Within level 'S5' of factor 'Soil'			
Groups	t	P(perm)	Sig	Groups	t	P(perm)	Sig
V1V3-V3V4	1.3052	0.2044	n.s.	V1V3-V3V4	1.046	0.407	n.s.
V1V3-V4V5	2.5964	0.0001	*	V1V3-V4V5	2.2573	0.0286	*
V1V3-V6V8	2.4604	0.0001	*	V1V3-V6V8	2.0213	0.0269	*
V3V4-V4V5	2.737	0.0001	*	V3V4-V4V5	2.2023	0.0295	*
V3V4-V6V8	2.3819	0.0292	*	V3V4-V6V8	1.975	0.0263	*
V4V5-V6V8	3.0994	0.0001	*	V4V5-V6V8	2.6036	0.0257	*
Within level 'S2' of factor 'Soil'				Within level 'S6' of factor 'Soil'			
Groups	t	P(perm)	Sig	Groups	t	P(perm)	Sig
V1V3-V3V4	1.1986	0.1417	n.s.	V1V3-V3V4	1.1846	0.2853	n.s.
V1V3-V4V5	2.5014	0.0298	*	V1V3-V4V5	2.2828	0.0309	*
V1V3-V6V8	2.1605	0.0284	*	V1V3-V6V8	2.0956	0.0299	*
V3V4-V4V5	2.4203	0.0001	*	V3V4-V4V5	2.4002	0.0252	*
V3V4-V6V8	1.9548	0.0325	*	V3V4-V6V8	2.028	0.0277	*
V4V5-V6V8	2.6996	0.0266	*	V4V5-V6V8	2.6875	0.0001	*
Within level 'S3' of factor 'Soil'				Within level 'S7' of factor 'Soil'			
Groups	t	P(perm)	Sig	Groups	t	P(perm)	Sig
V1V3-V3V4	1.4548	0.0629	n.s.	V1V3-V3V4	1.7998	0.0301	*
V1V3-V4V5	3.1867	0.0001	*	V1V3-V4V5	3.5139	0.0001	*
V1V3-V6V8	2.7774	0.0001	*	V1V3-V6V8	3.3066	0.0001	*
V3V4-V4V5	3.0202	0.0001	*	V3V4-V4V5	3.554	0.0001	*
V3V4-V6V8	2.472	0.0001	*	V3V4-V6V8	2.9953	0.0001	*
V4V5-V6V8	3.5613	0.0001	*	V4V5-V6V8	3.7117	0.0001	*
Within level 'S4' of factor 'Soil'				Within level 'S8' of factor 'Soil'			
Groups	t	P(perm)	Sig	Groups	t	P(perm)	Sig
V1V3-V3V4	1.2323	0.2828	n.s.	V1V3-V3V4	1.643	0.0293	*
V1V3-V4V5	2.4655	0.0293	*	V1V3-V4V5	3.057	0.0272	*
V1V3-V6V8	2.2073	0.0296	*	V1V3-V6V8	2.7799	0.0276	*
V3V4-V4V5	2.4128	0.0285	*	V3V4-V4V5	2.8625	0.0303	*
V3V4-V6V8	1.9763	0.0308	*	V3V4-V6V8	2.3204	0.0273	*
V4V5-V6V8	2.8774	0.0277	*	V4V5-V6V8	3.1919	0.0288	*

Supplementary Table S4. Multivariant PERMANOVA pairwise test. Determination of statistical differences between types of soil (n=4) in each 16S rRNA region. PRIMER e Permanova + (PRIMER-E Ltd, Plymouth, UK) was used in the implementation of the statistical analysis.

Soil samples: region*soil; factor:soil

Within level 'V1V3' of factor 'Region'				Within level 'V3V4' of factor 'Region'				Within level 'V4V5' of factor 'Region'				Within level 'V6V8' of factor 'Region'			
Groups	t	P(perm)	Sig	Groups	t	P(perm)	Sig	Groups	t	P(perm)	Sig	Groups	t	P(perm)	Sig
S1,S2	1.5328	0.0584	n.s.	S1,S2	1.4071	0.0555	n.s.	S1,S2	1.4993	0.0276	*	S1,S2	1.3807	0.0571	n.s.
S1,S3	2.8981	0.0001	*	S1,S3	2.647	0.0001	*	S1,S3	3.1556	0.0001	*	S1,S3	2.7741	0.0001	*
S1,S4	1.306	0.1169	n.s.	S1,S4	1.2392	0.1698	n.s.	S1,S4	1.2352	0.1685	n.s.	S1,S4	1.2882	0.1671	n.s.
S1,S5	1.9552	0.0001	*	S1,S5	1.9007	0.0293	*	S1,S5	2.2798	0.0001	*	S1,S5	2.101	0.0001	*
S1,S6	2.3372	0.0001	*	S1,S6	2.3212	0.0001	*	S1,S6	2.4083	0.0001	*	S1,S6	2.3841	0.0001	*
S1,S7	3.3893	0.0001	*	S1,S7	3.4104	0.0001	*	S1,S7	3.4774	0.0001	*	S1,S7	3.4077	0.0001	*
S1,S8	2.5314	0.0001	*	S1,S8	2.1841	0.0001	*	S1,S8	2.3312	0.0001	*	S1,S8	2.1991	0.0001	*
S2,S3	1.9501	0.0001	*	S2,S3	1.9824	0.0001	*	S2,S3	2.2798	0.0001	*	S2,S3	2.0679	0.0001	*
S2,S4	1.3324	0.0924	n.s.	S2,S4	1.3967	0.0601	n.s.	S2,S4	1.5403	0.0593	n.s.	S2,S4	1.5571	0.026	*
S2,S5	1.3982	0.079	n.s.	S2,S5	1.4701	0.0267	*	S2,S5	1.651	0.027	*	S2,S5	1.5888	0.0315	*
S2,S6	1.8235	0.0305	*	S2,S6	1.9581	0.0001	*	S2,S6	2.0017	0.0001	*	S2,S6	2.0126	0.0001	*
S2,S7	2.4323	0.0001	*	S2,S7	2.6286	0.0001	*	S2,S7	2.5714	0.0001	*	S2,S7	2.6518	0.0001	*
S2,S8	1.9544	0.0001	*	S2,S8	1.9386	0.0001	*	S2,S8	2.0443	0.0001	*	S2,S8	2.0333	0.0001	*
S3,S4	2.4941	0.0001	*	S3,S4	2.1233	0.0001	*	S3,S4	2.6556	0.0001	*	S3,S4	2.4211	0.0001	*
S3,S5	1.0359	0.4854	n.s.	S3,S5	0.95361	0.4606	n.s.	S3,S5	1.1917	0.1737	n.s.	S3,S5	1.0576	0.3416	n.s.
S3,S6	1.026	0.3756	n.s.	S3,S6	1.0096	0.3399	n.s.	S3,S6	1.1715	0.1687	n.s.	S3,S6	1.0704	0.348	n.s.
S3,S7	1.4004	0.0541	n.s.	S3,S7	1.3936	0.0611	n.s.	S3,S7	1.5912	0.0285	*	S3,S7	1.5277	0.0293	*
S3,S8	2.5598	0.0001	*	S3,S8	2.1698	0.0001	*	S3,S8	2.6837	0.0001	*	S3,S8	2.4136	0.0001	*
S4,S5	1.624	0.0001	*	S4,S5	1.5372	0.0555	n.s.	S4,S5	1.9191	0.0001	*	S4,S5	1.8386	0.0001	*
S4,S6	2.0015	0.0001	*	S4,S6	1.775	0.0292	*	S4,S6	1.9601	0.0001	*	S4,S6	1.9568	0.0001	*
S4,S7	3.0361	0.0001	*	S4,S7	2.8081	0.0001	*	S4,S7	2.9657	0.0001	*	S4,S7	3.1117	0.0001	*
S4,S8	2.3505	0.0001	*	S4,S8	2.0269	0.0001	*	S4,S8	2.176	0.0001	*	S4,S8	2.2206	0.0001	*
S5,S6	0.90069	0.5154	n.s.	S5,S6	0.95629	0.4262	n.s.	S5,S6	1.1165	0.261	n.s.	S5,S6	1.0638	0.286	n.s.
S5,S7	1.2881	0.1724	n.s.	S5,S7	1.3184	0.1417	n.s.	S5,S7	1.4221	0.0869	n.s.	S5,S7	1.4347	0.0865	n.s.

S5,S8	2.0329	0.0001	*	S5,S8	1.8333	0.0001	*	S5,S8	2.1249	0.0001	*	S5,S8	1.9932	0.0001	*
S6,S7	1.3963	0.119	n.s.	S6,S7	1.5084	0.0589	n.s.	S6,S7	1.7404	0.0001	*	S6,S7	1.6029	0.0001	*
S6,S8	2.3791	0.0001	*	S6,S8	2.2078	0.0001	*	S6,S8	2.4139	0.0001	*	S6,S8	2.3227	0.0001	*
S7,S8	3.0858	0.0001	*	S7,S8	2.6769	0.0001	*	S7,S8	2.6688	0.0001	*	S7,S8	2.8492	0.0001	*

Supplementary Table S5. Multivariant PERMANOVA pairwise test. Determination of statistical differences between 16S rRNA regions in each oral type (n= 5 for oral type 1, n=6 for oral type 2). and between each oral type within each 16S rRNA region (n= 20 for oral type 1, n=24 for oral type 2). PRIMER e Permanova + (PRIMER-E Ltd, Plymouth, UK) was used in the implementation of the statistical analysis.

Saliva samples: region*oral type; factor: region

Within level '1' of factor 'Oral type'				Within level '2' of factor 'Oral type'			
Groups	t	P(perm)	Sig	Groups	t	P(perm)	Sig
V1V3,V3V4	0.6948	0.6904	n.s.	V1V3,V3V4	0.9403	0.4932	n.s.
V1V3,V4V5	1.0724	0.5366	n.s.	V1V3,V4V5	1.2723	0.174	n.s.
V1V3,V6V8	0.75988	0.6663	n.s.	V1V3,V6V8	0.9811	0.4509	n.s.
V3V4,V4V5	0.83097	0.6388	n.s.	V3V4,V4V5	0.8964	0.5471	n.s.
V3V4,V6V8	0.78997	0.633	n.s.	V3V4,V6V8	1.0637	0.3605	n.s.
V4V5,V6V8	1.1409	0.5188	n.s.	V4V5,V6V8	1.3089	0.1437	n.s.

Saliva samples: region*oral type; factor: region

Within level 'V1V3' of factor 'Region'				Within level 'V4V5' of factor 'Region'			
Groups	t	P(perm)	Sig	Groups	t	P(perm)	Sig
O1,O2	1.6733	0.0001	*	O1,O2	1.6126	0.0001	*
Within level 'V3V4' of factor 'Region'				Within level 'V6V8' of factor 'Region'			
Groups	t	P(perm)	Sig	Groups	t	P(perm)	Sig
O1,O2	1.6186	0.0001	*	O1,O2	1.6943	0.0001	*
